# Programmable Macrophage Vesicle Based Bionic Self‐Adjuvanting Vaccine for Immunization against Monkeypox Virus

**DOI:** 10.1002/advs.202408608

**Published:** 2024-11-08

**Authors:** Weiqiang Lin, Chenguang Shen, Mengjun Li, Shengchao Ma, Chenxin Liu, Jialin Huang, Zuning Ren, Yuechao Yang, Minghai Zhao, Qiulin Xie, Shuang Guo, Wei Wang, Kaiyuan Wang, Qiang Ma, Yideng Jiang, Judun Zheng, Yuhui Liao

**Affiliations:** ^1^ NHC Key Laboratory of Metabolic Cardiovascular Diseases Research, Ningxia Key Laboratory of Vascular Injury and Repair Research Ningxia Medical University Yinchuan 750004 P. R. China; ^2^ Institute for Engineering Medicine Kunming Medical University Kunming 650500 P. R. China; ^3^ BSL‐3 Laboratory (Guangdong), Guangdong Provincial Key Laboratory of Tropical Disease Research, School of Public Health Southern Medical University Guangzhou 510515 P. R. China; ^4^ School of Laboratory Medicine and Biotechnology Southern Medical University Guangzhou 510515 P. R. China; ^5^ Molecular Diagnosis and Treatment Center for Infectious Diseases Dermatology Hospital, Southern Medical University Guangzhou 510091 P. R. China; ^6^ Departments of Diagnostic Radiology, Surgery, Chemical and Biomolecular Engineering, and Biomedical Engineering, Yong Loo Lin School of Medicine and College of Design and Engineering National University of Singapore Singapore 119074 Singapore

**Keywords:** bionic vaccine, extracellular enveloped virion, intracellular mature virion, monkeypox virus, macrophage vesicles

## Abstract

The emergence of monkeypox has become a global health threat after the COVID‐19 pandemic. Due to the lack of available specifically treatment against MPV, developing an available vaccine is thus the most prospective and urgent strategy. Herein, a programmable macrophage vesicle based bionic self‐adjuvanting vaccine (AM@AEvs‐PB) is first developed for defending against monkeypox virus (MPV). Based on MPV‐related antigen‐stimulated macrophage‐derived vesicles, the nanovaccine is constructed by loading the mature virion (MV)‐related intracellular protein (A29L/M1R) and simultaneously modifying with the enveloped virion (EV) antigen (B6R), enabling them to effectively promote antigen presentation and enhance adaptive immune through self‐adjuvant strategy. Owing to the synergistic properties of bionic vaccine coloaded MV and EV protein in defensing MPV, the activation ratio of antigen‐presenting cells is nearly four times than that of single antigen in the same dose, resulting in stronger immunity in host. Notably, intramuscular injection uptake of AM@AEvs‐PB demonstrated vigorous immune‐protective effects in the mouse challenge attempt, offering a promising strategy for pre‐clinical monkeypox vaccine development.

## Graphical abstract

The monkeypox‐specific bionic vaccine (AM@AEvs‐PB) is consists of IMV antigens (A29L, M1R), the EEV antigen (B6R), and MPV‐preactivated macrophagederived vesicles. AM@AEvs‐PB can induce enhanced innate immune responses, promote cross‐presentation of antigens to dendritic cells (DCs), and elicit robust adaptive immune responses, realizing immunization protection against Monkeypox Virus.

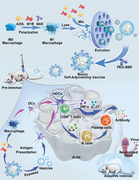

## 1 Introduction

Monkeypox (Mpox), caused by the zoonotic monkeypox virus (MPV), has spread to more than 117 countries worldwide through physical contact with infected individuals, animals, or contaminated materials, posing a significant threat to global public health.^[^ ^1^ ^]^ Currently, the supportive care and pain control are suggested for the Mpox patients clinically, and the pills for smallpox (such as tecovirimat, brincidofovir, and vaccinia immune globulin) have been developed for use in Mpox patients in the certain situations, but the available specifically treatment against MPV is still lack. Compared to the specific medicines, developing an available vaccine is more prospective. Live‐attenuated vaccines have been widely used for preventing infectious diseases owing to its broad protection and rapid industrial production, but high‐level biological safety environment is needed during production.^[^ ^2^ ^]^ JYNNEOS and ACAM2000 are two live‐attenuated vaccines used during the Mpox outbreak in America on May 2022, though both performed a certain protective activity against MPV due to the cross‐immunity of smallpox virus and MPV, the specificity is still limited.^[^ ^1c^ ^]^ Message RNA (mRNA) vaccines, as highly specific vaccines, have emerged as the cornerstone of next‐generation vaccination strategies, but there remained challenges pertaining to the easy degradation the efficient delivery of mRNA within the body and the stringent storage condition.^[^ ^3^ ^]^ Protein subunit vaccines contain viral or pathogenic proteins, can not only offer effective and specific immunity protection without causing obvious negative effects,^[^ ^4^ ^]^ but also be lyophilized and stored at room temperature for extended periods, making them promising candidates as the MPV vaccines.^[^ ^5^ ^]^

Protein subunit vaccines require defined immunogens and immune adjuvants to induce immunity against the virus or pathogen.^[^ ^6^ ^]^ While selecting immunogenic candidates for Mpox, the type of MPV antigen and its immune‐protective efficacy need to be considered. MPV contains the extracellular enveloped virion (EEV) and intracellular mature virion (IMV) particles during its infection and replication cycles, which are released from infected host cells by exocytosis or lysis in the early or late stages of infection, respectively.^[^ ^7^ ^]^ EEV is responsible for intercellular transport and binds to host cell membranes in a non‐pH‐dependent manner, while IMV is more stable and responsible for host‐to‐host transport.^[^ ^8^ ^]^ Thus, IMV and EEV membrane proteins are suitable for constructing recombinant protein vaccines. Among all the IMV antigens and EEV antigens, A29L (IMV‐specific antigen), M1R (IMV‐specific antigen) and B6R (EEV‐specific antigen) are well known as effective neutralizing antibody targets for Mpox. In addition, the combination of IMV‐ and EEV‐specific immunogens has been found to provide more protection than either immunogen alone.^[^ ^9^ ^]^ Thus, these three antigens are selected and combined together to protect against MPV in our study. Effective vaccine often relies on the addition of immune adjuvants, such as aluminum salts and Toll‐like receptor agonists CpG, which can enhance the strength and duration of innate immunization, especially when high‐performance antigens have not been identified.^[^ ^10^ ^]^ An ideal adjuvant can not only effectively deliver target molecules to achieve specific and long‐lasting humoral and cellular immune responses, but also avoid rapid clearance from the body. However, there is a lack of adjuvants for Mpox vaccines that possess both delivery and immune‐enhancing functionalities.

Nanovaccines have recently attracted extensive attention because of their unique multifunctionality, including specific delivery, lymph node accumulation, and fewer side effects, making them promising for infectious disease prevention^[^ ^11^ ^]^ and cancer therapy.^[^ ^12^ ^]^ Biomimetic nanovaccines possess similar properties as pathogens including shape, size, and charge,^[^ ^13^ ^]^ which can be facilitated to effectively improve their performance by enhancing antigen‐presenting cells (APC) uptake.^[^ ^14^ ^]^ Different from the live‐attenuated vaccines, the functional antigens would be selected when designing and fabricating the nanovaccines. Several studies have shown that bionic extracellular vehicles (Evs) can improve the stability of the antigen‐adjuvant co‐system while enhancing uptake efficiency via vesicle‐APC adhesive interactions.^[^ ^15^ ^]^ Pre‐activated macrophage Evs specifically engineered against pathogens exhibit high expression levels of specific pathogenic surface receptors and enhance the adjuvant effect with minimal immune rejection.^[^ ^16^ ^]^ Despite these advancements, there is still a lack of bionic nanovaccines for MPV prevention.

**Scheme 1:**
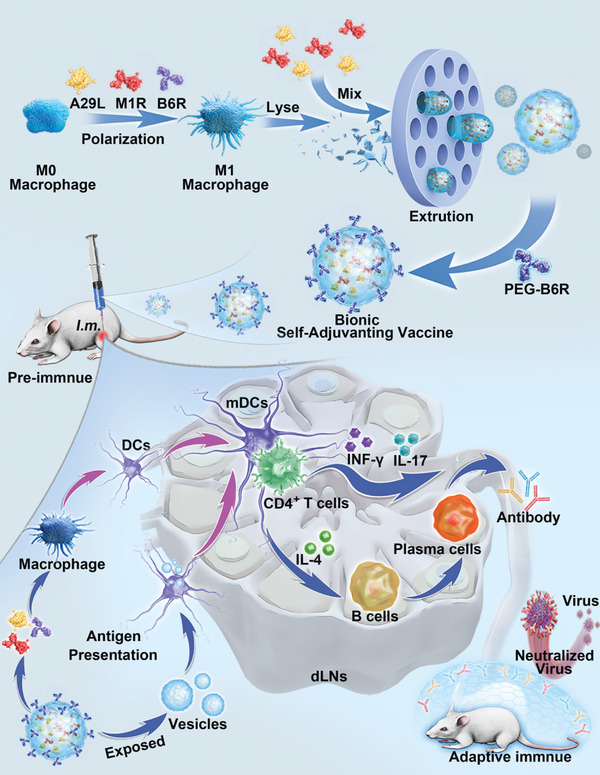
Schematic illustration of programmable macrophage vesicle simulating bionic self‐adjuvanting vaccine for immunization against MPV.

Herein, considering the advantage of the nanovaccine, we developed a self‐adjuvant bionic vaccine to protect against MPV (**Scheme** **1**). This bionic vaccine (AM@AEvs‐PB) contains IMV antigens (A29L, M1R) and the EEV antigen (B6R) MPV with programable macrophage‐derived vesicles. The vaccine effectively induces innate immune responses, promotes cross‐presentation of antigens to dendritic cells (DCs), and elicits robust adaptive immune responses, including the proliferation of germinal center B cells, development of MPV‐specific CD4^+^ T‐cell and IgG responses, and induction of antigen‐specific memory responses. Furthermore, the protein capsid‐like vaccine protected mice against infection by inducing a notable immune response and neutralizing the virus after the challenge (Scheme 1). Our findings represent a promising and novel approach for safeguarding against an emerging MPV infection.

## 2 Results

### 2.1 Characterization and Transcriptomic Analysis of MPV‐Related Antigen‐Activated Macrophages

**Figure 1:**
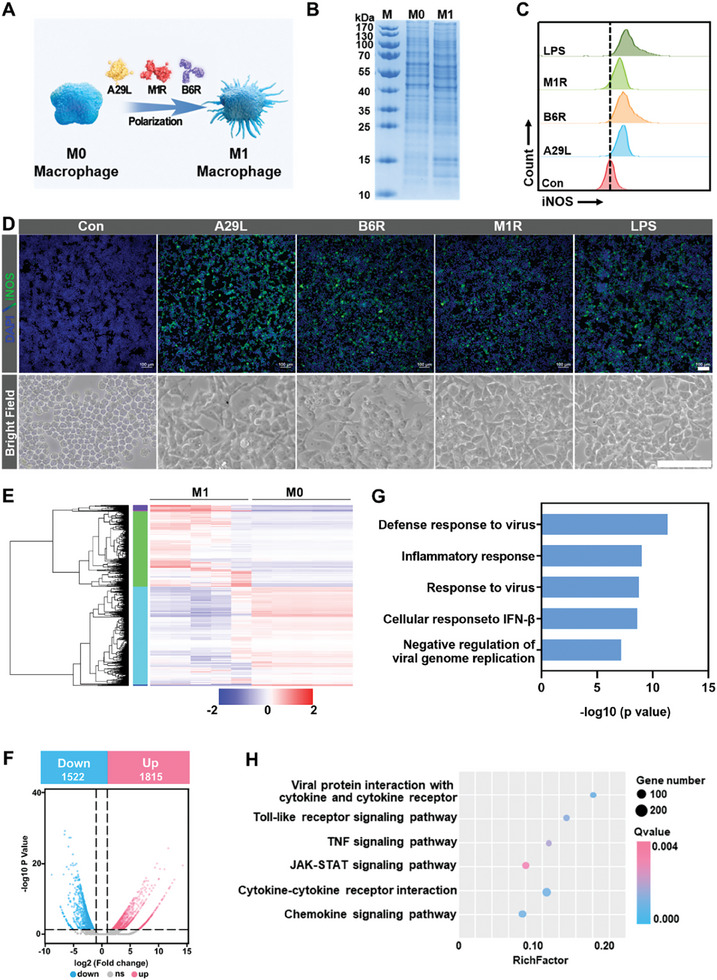
Characterization and transcriptomic analysis of MPV‐related antigen‐activated macrophages. A) A scheme illustrating the activation of RAW 264.7 cells by antigens (A29L, B6R, and M1R). B) The SDS‐PAGE of RAW 264.7 cells before (M0) and after activation (M1). The analysis of iNOS after treating with different antigens by C) flow cytometry and D) immunofluorescence staining (green fluorescence for iNOS and blue fluorescence for DAPI), and shape change of RAW 264.7 cells were observed in bright field. (Scale bar: 100 µm). E) Heatmap of differentially expressed genes between the M1 and M0 macrophages. F) The volcano plots of genes detected by RNA‐Seq, G) the KEGG analysis and H) the GO analysis of activated RAW 264.7 cells.

Macrophages act as sentinels of the innate immune system and protect against viruses by phagocytosing them, processing antigens, and secreting cytokines that activate adaptive immune responses, including humoral and cellular immune responses.^[^ ^17^ ^]^ Activated macrophages express high levels of specific receptors to target pathogens and enhance adjuvant effects.^[^ ^16^, ^18^ ^]^ Thus, the activation of macrophages was a critical preliminary step for bionic vaccine construction. IMV antigens (A29L, M1R) and EEV (B6R) were introduced into the macrophages to determine their effectiveness for macrophage activation (**Figure** **1**A). This activation was confirmed by noticeable changes in protein expression within the 10–15 kDa, which may be related to specific receptors involved in the immune response (Figure 1B). Additionally, iNOS, the biomarkers marker for M1 polarization, showed a significant increase in expression, compared to the control group, during flow cytometry and immunofluorescence staining analyses, indicating the efficacy of the antigens A29L, B6R, and M1R for successful macrophage activation (Figure 1C; Figure S1, Supporting Information). The higher level of iNOS expression and discernible morphological changes in macrophages (Figure 1D, Figure S2, Supporting Information). Moreover, the activated proportion of macrophages demonstrates a dose‐dependent augmentation upon exposure to escalating antigen concentrations, and the proportions leveled off at the dose of 2 µg, so we chose it as the optimal dose (Figure S3, Supporting Information). These results confirm the successful introduction of agents that induce macrophage activation and subsequent M1 polarization.

The effects of MPV antigens on macrophage cells were investigated via the RNA sequencing of RAW 264.7 cell lines. The analysis revealed 1815 upregulated and 1522 downregulated genes in the antigen‐treated group compared to the control group (Figure 1E,F, Supporting Information). Gene ontology annotations highlighted enrichment in antiviral defense, inflammatory responses, viral responses, and cellular responses to interferon‐β among differentially expressed genes (Figure 1G). Moreover, Kyoto Encyclopedia of Genes (KEGG) and Genomes analysis (GO) indicated enrichment in pathways involving viral proteins with cytokines and cytokine receptors, Toll‐like receptor signaling, TNF signaling, and chemokine signaling (Figure 1H; Table S1, Supporting Information). RNA sequencing analysis validated the activation of Toll‐like receptor signaling and inflammatory factor receptor pathways (cytokines and cytokine receptors, TNF signaling, and chemokine signaling), and the findings were in accordance with the iNOS results.

The role of NLRP3 inflammasome activation in antigens‐stimulated macrophage during the pre‐activation process was first explored. Our findings revealed that the antigens triggered upregulation of caspase‐1, NLRP3 and NF‐κB protein expression (Figure S4, Supporting Information). Furthermore, macrophage activation was assessed by immunofluorescence detection of iNOS, IL‐6, and TNF‐α biomarkers. Notably, a significant increase in expression levels was observed in the antigen groups (A29L, B6R, and M1R) relative to the control group (Figure S5, Supporting Information), confirming the potency of these antigens in activating macrophages. In short, integrating the RNA‐seq data, our results suggest that the antigens activate macrophages via the NLRP3 inflammasome pathway, underscoring the capacity of MPV antigens to initiate macrophage‐mediated innate immune responses against the virus. These findings suggest the pre‐activation of macrophages by MPV antigens, forming the foundation of our bionic vaccine.

### 2.2 Rational Design, Synthesis, and Characterization of AM@AEvs‐PB

**Figure 2:**
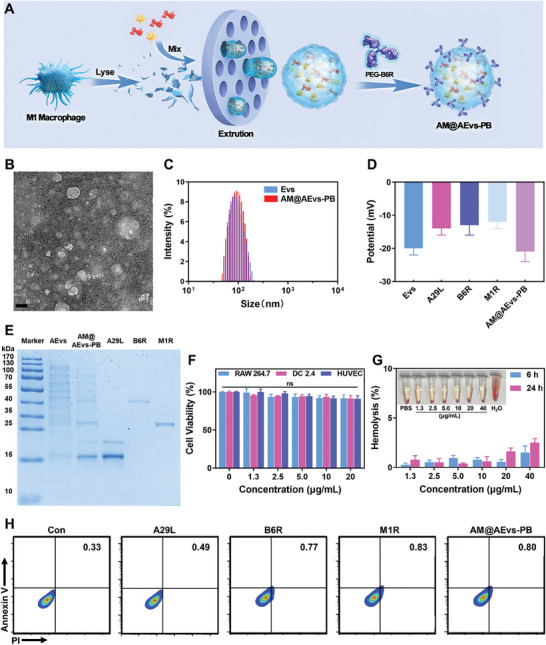
Rational design, synthesis, and characterization of AM@AEvs‐PB. A) Schematic illustration of the rational design of AM@AEvs‐PB. B) Representative TEM images of AM@AEvs‐PB (scale bar: 100 nm). C) Size distribution and D) Zeta potential detection via DLS. E) SDS‐PAGE of the components. F) CCK8 assays and G) hemolytic rate after treating with. AM@AEvs‐PB. H) Flow cytometry of antigens and AM@AEvs‐PB on macrophage apoptosis rate (Anexinn‐V PI/FITC). Data are shown as mean ± SD. Differences with *P* < 0.05 (*), *P* < 0.01 (**) or *P* < 0.001 (***) were considered statistically significant, and ns means no significance.

As reported previously, the bionic engineered vesicles have the natural complex characteristics of the source cells.^[^ ^19^ ^]^ We aim to fabricate a bionic vaccine based on engineering vesicles derived from pre‐activated macrophages, which have been stimulated the antiviral pathways and inflammatory pathways, to overcome limitations of immunogenicity and clearance in recombinant protein vaccines. To prepare AM@AEvs‐PB, pre‐activated macrophages were lysed and formed activated vesicles (AEvs), which were then loaded with IMV antigens A29L and M1R. Afterwards, the vesicles were conjugated with PEGylation B6R (**Figure** **2**A). The characterization of naive AEvs and AM@AEvs‐PB through transmission electron microscopy (TEM) showed that both had similar spherical shapes and an average size of ≈100 nm (Figure S6, Figure 2B, Figure S7, Supporting Information). The dynamic light scattering revealed a hydrodynamic size distribution peaking at ≈100 nm for AEvs and AM@AEvs‐PB (Figure 2C), with a surface charge of ≈ ‐20 mV (Figure 2D), which was similar with the size and negative charge of MPV. As shown in the Figure 2E, the presence of A29L, B6R, and M1R antigens in AEvs was confirmed according to SDS PAGE image, and the loading efficacy were 20% (A29L), 27% (B6R), and 19% (M1R) according to the standard curve (Figure S8, Supporting Information). These results establish the successful fabrication of AM@AEvs‐PB.

Stability, as an important property of nano‐vaccine, was evaluated via observing the size changes of AM@AEvs‐PB in PBS and FBS for 7 days. As displayed in Figure S9 (Supporting Information), the size of the bionic vaccine stayed relatively constant, illustrating macrophages derived vesicles vaccine AM@AEvs‐PB possess remarkable stability. Besides, the biocompatibility of the vaccine is crucial for future development. The CCK8 assay and hemolysis assay were performed to assess the cytotoxicity and hemolytic potential of AM@AEvs‐PB to evaluate the biocompatibility of the vaccine, respectively. Different cell lines including RAW 264.7, DC 2.4, and HUVEC were analyzed. The CCK8 results in Figure 2F indicated that AM@AEvs‐PB exhibited minimal cytotoxicity in all cell lines tested at concentrations up to 20 µg mL^−1^. Furthermore, the hemolytic rate remained below 5% even after treating the cells with various AM@AEvs‐PB concentrations for 24 h (Figure 2G). Moreover, apoptosis analysis (Figure 2I; Figure S10, Supporting Information) showed a negligible increase in the apoptosis rate with an approximate majority of macrophages (≈0.85%) after 24‐h exposure to AM@AEvs‐PB compared with the control groups, confirming the non‐cytotoxicity of our bionic vaccine.

Regarding potential systemic toxicity, Balb/c mice were intramuscularly injected (i.m.) with antigen (A29L, B6R, and M1R) and the bionic vaccine, followed by H&E staining of harvested organs. As illustrated in Figure S11 (Supporting Information), there was no noticeable acute organ damage in the AM@AEvs‐PB group when compared to the control group and antigen groups, underscoring the safety of the AM@AEvs‐PB vaccine. Besides, the endotoxin level of the vaccine was also acceptable (Figure S12, Supporting Information). Taken together, the bionic vaccine AM@AEvs‐PB is substantiated well‐constructed with the high biocompatibility in vitro and in vivo.

### 2.3 Internalization of Bionic Vaccine In Vitro and Retention In Vivo

**Figure 3:**
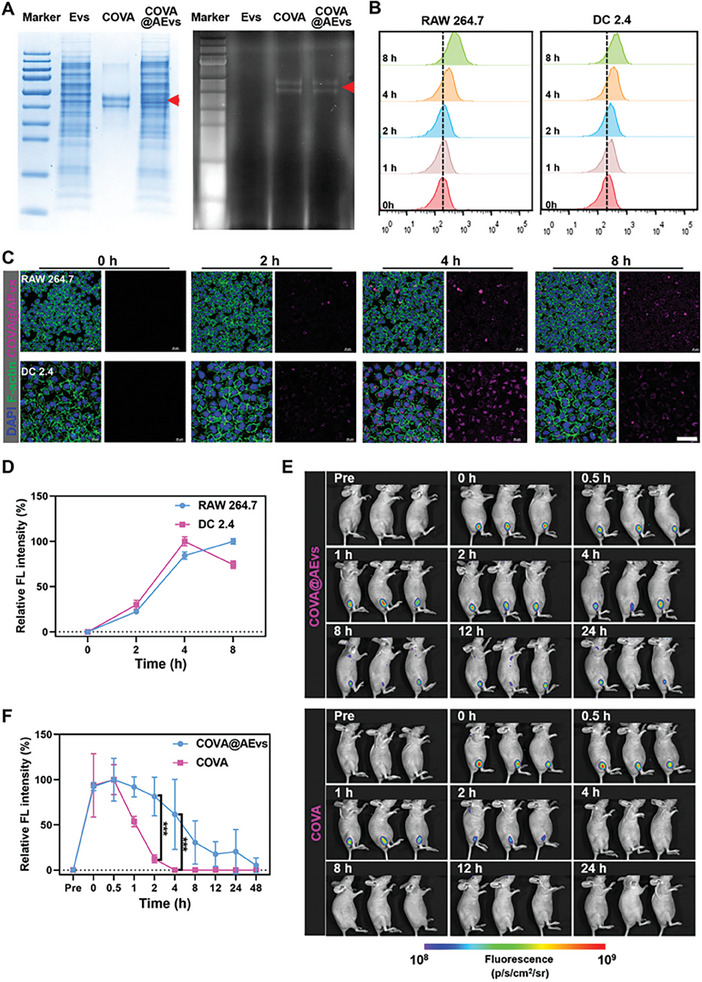
Internalization of biomimetic bionic vaccine in vitro and retention in vivo. A) SDS PAGE and fluorescence PAGE of COVA@AEvs; B) Cellular uptake of COVA@AEvs by RAW 264.7 and DC 2.4 cells; C) Intracellular uptake images of uptake at different time points (0, 2, 4, and 8 h) and D) is the analysis of relative fluorescence intensity. E) The fluorescence images after I.m injection with COVA@Evs and COVA, and F) is relative fluorescence intensity analysis. Data are shown as mean ± SD. Differences with *P* < 0.05 (*), *P* < 0.01 (**), or *P* < 0.001 (***) were considered statistically significant.

The maturation and further presentation of APCs are crucial for effective vaccination, and it is essential for AM@AEvs‐PB to be recognized and internalized for maturing APCs and effectively eliciting immune responses. As the lack of fluorescence labeled MPV antigens, the antigens were substituted by Cy5‐labeled ovalbumin (COVA), which possesses a comparable zeta potential as the antigens (Figure S13, Supporting Information), to examine the cellular uptake in vitro. Initially, SDS‐PAGE gel analysis was performed to confirm the loading of COVA in the vesicles. As depicted in **Figure** **3**A, COVA@AEvs produced a band similar to AEvs alone (left) and exhibited a consistent fluorescent band with COVA (right), providing evidence for the successful loading of COVA. Additionally, we calculated a loading rate of 17% for the COVA‐loaded vesicles utilizing a standard curve (Figure S14, Supporting Information).

To evaluate the cellular uptake, we co‐cultured COVA@AEvs with RAW 264.7 cells and DC 2.4 cells for various time intervals (ranging from 0 to 8 h). Flow cytometry analysis indicated that macrophages displayed enhanced internalization compared to dendritic cells at the 8‐hour time point, whereas DCs demonstrated faster uptake initially (Figure 3B). Confocal laser scanning microscopy (CLSM) confirmed these results (Figure 3C,D). Interestingly, the fluorescence intensity began to decrease in DC 2.4 cells after peaking at 4 h post co‐incubation, whereas RAW 264.7 cells exhibited an increase in fluorescence intensity over time. This suggests that macrophages, possessing a higher antigen presentation capacity and homology affinity of vesicles, exhibit more efficient uptake by dendritic cells compared to RAW 264.7 cells. In addition, internalization of COVA in both cell lines was determined (Figure S15, Supporting Information), revealing that free COVA demonstrated slower uptake by cells compared to encapsulated COVA. In short, COVA@AEvs possesses desirable cellular uptake properties because vesicles enhance uptake efficiency.

Prolonged antigen retention enhances APC maturation, promoting a longer‐lasting innate immune response in the body.^[^ ^20^ ^]^ The retention time of COVA@AEvs and free COVA was assessed in vivo to examine whether vesicles could extend antigen retention following intramuscular injection. Though the amount of COVA and COVA@AEvs for i.m. were consistent (Figure S16, Supporting Information). A noticeable disparity in fluorescence decay was observed at the injection site over a 48‐hour observation period (Figure 3E; Figure S17, Supporting Information). While free COVA exhibited rapid decline and faded after 4 h, COVA@AEvs was sustained for ≈24 h in the treated mice. Further analysis revealed a significant difference in retention time between the two groups at 2 h post‐injection (Figure 3F). In order to further evaluate the biological functions in vivo, the organ distribution of the macrophage vesicles‐based vaccine was studied by the ex vivo fluorescence imaging. Intense fluorescence signals of COVA@AEvs were observed in the liver tissues (Figure S18, Supporting Information), indicating that the nanovaccine was preferred to selectively metabolized in liver own to reticuloendothelial system, which is coincides with the general metabolism of vaccine via intramuscular injection.^[^ ^21^ ^]^ Based on our findings in the alternative model COVA@AEvs, we discovered that vesicles as carriers not only enhanced cellular uptake but also prolonged antigen retention. Thus, we hypothesize that our AM@AEvs‐PB exhibits more robust cellular uptake and prolonged antigen retention compared to the antigen alone attributing to the presence of AEvs.

### 2.4 Innate Immune Responses Induced by AM@AEvs‐PB In Vitro

**Figure 4:**
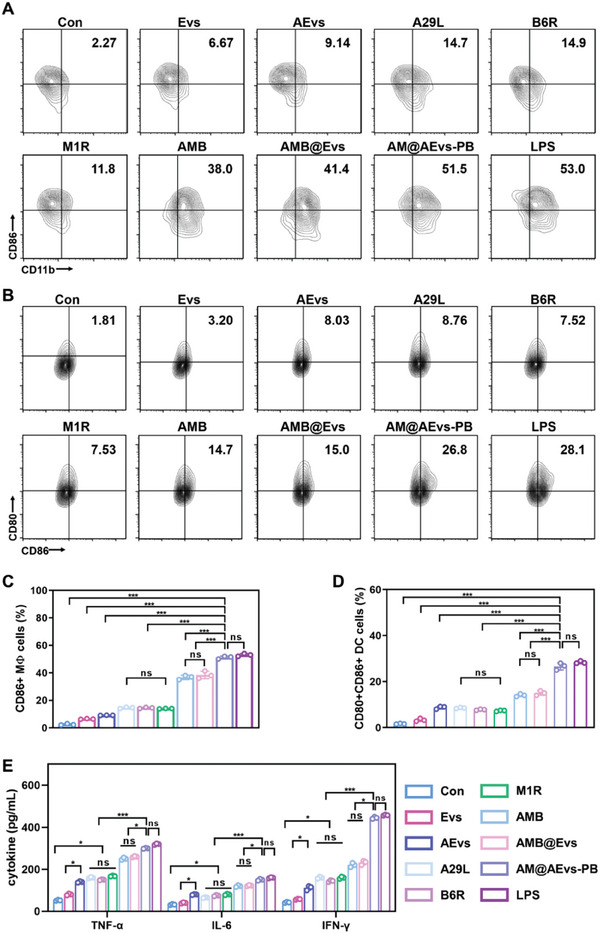
The immunogenicity of AM@AEvs‐PB in vitro. A) In vitro activation of BMDMs and B) BMDCs by Evs, AEvs, A29L, B6R, M1R, AMB@Evs, AM@AEvs‐PB, and LPS. C) and D) are statistical analysis respectively. E) Secretion of TNF‐α and IL‐6 from BMDMs and IFN γ from BMDCs after stimulation. Data are shown as mean ± SD. Differences with *P* < 0.05 (*), *P* < 0.01 (**), or *P* < 0.001 (***) were considered statistically significant, and ns means no significance.

Since the vesicle‐based bionic system demonstrates superior cellular uptake and prolonged antigen retention, we postulated that pre‐activation induced innate immune responses. To investigate the immunogenicity of AM@AEvs‐PB, the activation and maturation of APCs were examined by evaluating co‐stimulatory marker expression. The bone marrow‐derived macrophages (BMDMs) and bone marrow‐derived dendritic cells (BMDCs) were used as appropriate models for in vitro immunogenicity studies. After deriving and culturing BMDMs and BMDCs as reported, we co‐incubated the cells with vesicles derived from various sources, including M0 macrophage‐derived vesicles (Evs), AEvs, A29L, B6R, and M1R, a tri‐antigen complex (AMB), Evs‐loaded AMB (AMB@Evs) and AM@AEvs‐PB. Lipopolysaccharide (LPS) stimulation was included as a positive control. When AM@AEvs‐PB was administered to BMDMs, it had a substantial synergistic effect on the upregulation of co‐stimulatory markers, including CD11b and CD86, compared to other groups (**Figure** **4**A,C), indicating the bionic vaccine can effectively trigger the activation the macrophage. Notably, the presence of AEvs along with the antigen resulted in a greater population of CD11b^+^CD86^+^ BMDMs compared to Evs. Additionally, the percentage of CD11b^+^CD86^+^ BMDMs in the AMB sample was significantly higher than in single antigen groups (A29L, B6R, and M1R). Obviously, the proportion of activated BMDMs in AMB@Evs remained similar to AMB, while the stimulation of BMDMs with AM@AEvs‐PB led to a substantial enrichment of CD11b^+^CD86^+^ cells (51.5%), resembling the percentage in the LPS group (53%). These findings suggest that AEvs acted as an adjuvant in the bionic system. A similar upregulation of co‐stimulatory markers, such as CD80 and CD86, was also detected in BMDCs upon stimulation (Figure 4B,D), announcing that AM@AEvs‐PB can also induce the maturation of DCs, which is crucial to the antigen presentation and adaptive immune cell recruitment. In a word, AEvs enhance immnunogenicity of the antigens as adjuvant, which together make up AM@AEvs‐PB to synergistically strengthen the activation and maturation of APCs.

The induction of inflammatory cytokines, a component of innate immunity, was assessed following the administration of cells in vitro. Comparative analysis with the control and Evs groups revealed notable enhancements in the secretion of TNF‐α and IL‐6 from BMDMs in response to AEvs, individual antigens (A29L, B6R, and M1R), AMB, AMB@Evs, and AM@AEvs‐PB. Notably, the elevated levels of TNF‐α and IL‐6 secretion were observed in the AM@AEvs‐PB group, indicating the substantial positive effect of this system on inflammatory responses compared to other groups (Figure 4E). Additionally, AM@AEvs‐PB substantially promoted IFN‐γ secretion in BMDCs (Figure 4E), which is essential for the antiviral and immunoregulatory activities.

The previous researches have reported that pathogen and virus infection, self‐DNA damage and tumor DNA are the three key factors causing the activation of cGAS‐STING signaling.^[^ ^22^ ^]^ Among them, most of DNA viruses, such as human cytomegalovirus (HCMV), herpes simplex virus type 1 (HSV‐1), and poxviruses can activate the cGAS‐STING cytosolic DNA‐sensing pathway and initiate an antiviral immune response. Especially, monkeypox is a typical kind of DNA poxviruses, which cause human and veterinary diseases.^[^ ^23^ ^]^ Building on this, our study examines the vesicles‐based vaccine's potential to enhance immune responses via the cGAS‐STING pathway. Our findings, as depicted in Figures S19 and S20 (Supporting Information), the vesicles‐based vaccine triggered the upregulation of cGAS and STING protein expression in both BMDM and BMDC cells compared to the control group, suggesting the activation of cGAS‐STING pathway by the vessicles. Consequently, our data suggest that macrophage‐derived vesicles function as adjuvants, leveraging the cGAS‐STING pathway to augment immune responses against viral infections. Importantly, this heightened inflammatory environment facilitates the recruitment and activation of adaptive immune cells.

### 2.5 Systemic Immune Responses after Vaccination with AM@AEvs‐PB

**Figure 5:**
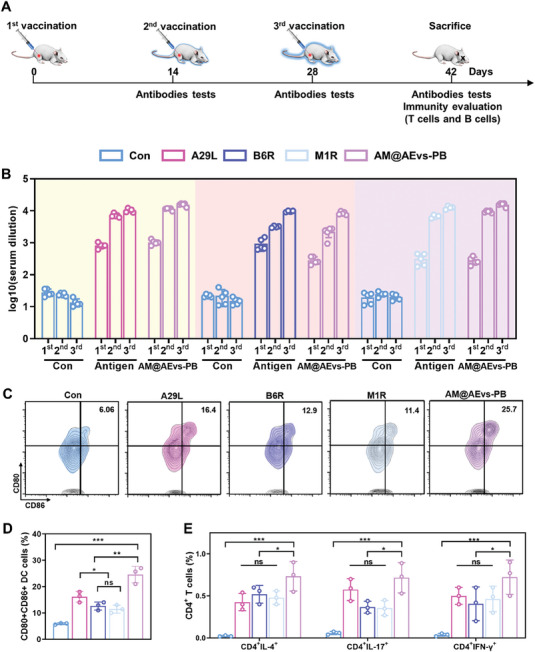
Systemic immune responses after vaccination with AM@AEvs‐PB. A) Schematic of immunization in mice. B) Changes in antibodies titers after immunization. C) Activation of splenic DC cells after the last immunization of mice, and D) is the analysis. E) Changes in the proportion of IL‐4, IFN‐γ and IL‐17‐screting CD4^+^ T cells. Data are shown as mean ± SD. Differences with *P* < 0.05 (*), *P* < 0.01 (**), or *P* < 0.001 (***) were considered statistically significant, and ns means no significance.

As our constructed bionic system has efficiently initiated immune response, we next evaluate the subsequent humoral and adaptive responses after vaccination. The vaccines or antigen were administered via i.m. thrice, 2 weeks apart, with each administration occurring 2 weeks apart. The mice received a dose of 6 µg of antigen in each group. Antibodies in mouse sera were assessed at 2 weeks post‐vaccination to evaluate the humoral immune response (**Figure** **5**A). Two weeks after administering the third vaccine, mice injected with antigens and AM@AEvs‐PB exhibited a significant IgG response post‐injection compared to the control group, indicating an enhanced effect on humoral immunity. Besides, the IgG titers of A29L, B6R, and M1R in the AM@AEvs‐PB group were higher than those in single antigen groups, which suggested a more robust immune response in the bionic vaccine group (Figure 5B). In addition, we also assessed the specificity of the bionic vaccine by replacing the MPV‐related antigens with COVID‐19‐related antigen RBD. Compared with mice in AM@AEvs‐PB group, the replaced RBD vaccine induce similar MPV‐specific titer as the control group (Figure S23, Supporting Information), indicating the specificity of our constructed vaccine. The DC, CD8^+^, and CD4^+^ T‐cell responses were also evaluated in vaccinated mice. AM@Evs‐PB administration notably increased CD80^+^CD86^+^ DC numbers compared to other control groups, confirming the maturation of DCs, which is beneficial for subsequent T‐cell activation (Figure 5C,D).

Splenic CD4^+^ and CD8^+^ T‐cell responses were evaluated in immunized mice. Cytokine‐secreting effector T cells, including IL‐4, IFN‐γ, and IL‐17‐secreting cells, were identified via flow cytometry. IL‐4 can reportedly promote the activation of B cells, an essential component of humoral immunity. As shown in Figure 5E and Figure S25 (Supporting Information), the mice in the AM@AEvs‐PB group had elevated IL‐4‐secreting CD4^+^ T cells compared to those in single antigen groups (A29L, B6R, and M1R) and the control group. We separately evaluated IL‐17 and IFN‐γ‐secreting CD4^+^ T cells, which were associated with inflammatory responses and antiviral functions, respectively. Compared with mice vaccinated with individual antigens, those immunized with the bionic vaccine showed an increased number of IL‐17‐secreting (Figure 5E; Figure S26, Supporting Information) and IFN‐γ‐secreting (Figure 5E; Figure S27, Supporting Information) CD4^+^ T cells.

Additionally, granzyme B and perforin‐secreting CD8^+^ T cells were also analyzed, and both showed a significant difference in mice of AM@AEvs‐PB group compared to control group (Figure S29, Supporting Information). As the Th1/Th2 balance is crucial to ensure a comprehensive assessment of the immune response elicited by the vaccine, we conducted the Th1/Th2 phenotype by analyzing the cytokine‐secreting effector T cells. Compared with mice vaccinated with individual antigens, those immunized with the bionic vaccine showed an increased number of IFN‐γ‐secreting and IL‐4‐secreting CD4^+^ T cells, which represented the Th1 cells and Th2 cells separately. suggesting the bionic vaccine could induce an antigen‐specific CD4^+^ T cell response biased toward mixed Th1/Th2 phenotype.

Since germinal center (GC) reactions are essential for inducing antibody responses and humoral immunity, we assessed the antigen‐mediated activation of GC B cells. GCs were identified based on their high expression of Fas and N‐glycolylneuraminic acid (probed using the GL7 antibody) using flow cytometry. The analysis of draining lymph nodes (dLNs) in immunized mice revealed that ≈2.5% of B220^+^ B cells were GL7^+^ Fas^+^ GC B cells in the AM@AEvs‐PB group. This represented a 3.9‐fold, 2.13‐fold, and 3.5‐fold increase in the number of GC‐positive cells compared to the A29L, B6R, and M1R groups, respectively (Figure S31, Supporting Information). These results suggested that elevated AM@AEvs‐PB‐induced protective cellular responses and humoral immune responses, indicating the migration of APCs from the injected sites to the lymph nodes.

Moreover, in order to evaluate the efficacy of vaccine in different genetic background, we immunized C57 mice and Balb/c mice meanwhile, and detected the and T cells response and specific titer by flow cytometry and ELISA separately. As illustrated in Figure S32 and S33 (Supporting Information), no significant difference in CD4^+^ T cells and CD8^+^ T cells could be found in two kinds mice. Furthermore, similar MPV‐specific titers could be observed in both Balb/c mice and C57 mice (Figure S34, Supporting Information). Taken together, our vaccine has a good effect on mice with different genetic backgrounds, and the efficacy needs to be further evaluated in humanized mice and non‐human primates.

### 2.6 Efficient Neutralizing of the Virus by AM@AEvs‐PB

**Figure 6:**
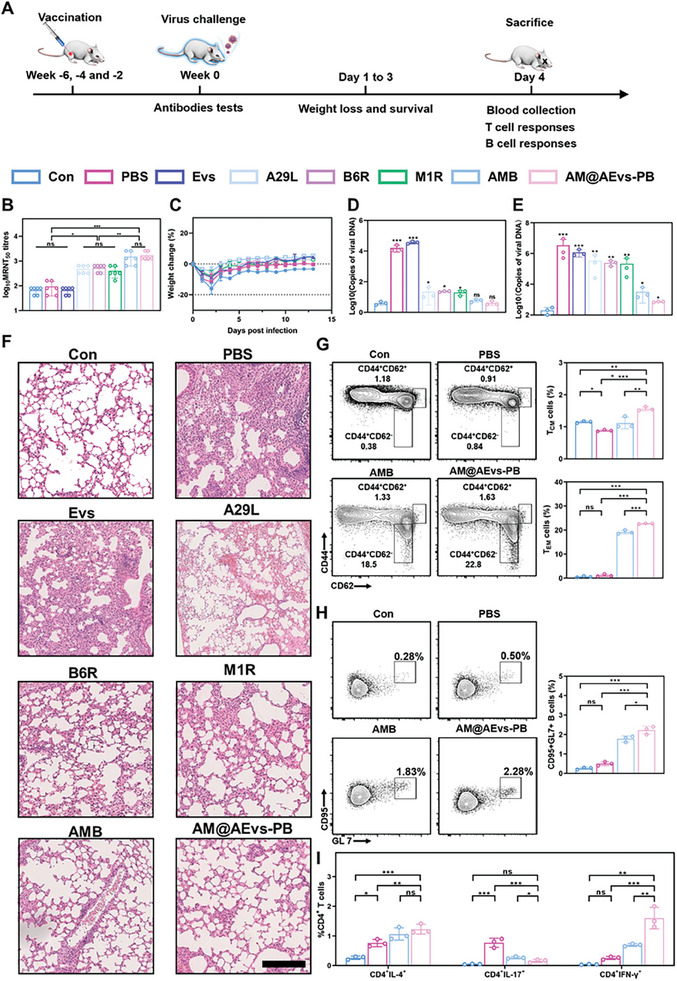
AM@AEvs‐PB efficiently neutralizes the VACA virus. A) Schematic illustration; B) MRNT_50_ value C) body weight of different groups of mice (Con, PBS, Evs, A29L, B6R, M1R, AMB, and AM@AEvs‐PB). Viral mRNA copies in D) serum and E) lung via qPCR. F) HE staining of the lung (Scale bar: 200 µm). Changes in the proportion of G) T_CM_ and T_EM_ in CD4^+^ T cells, and H) GC B cells. I) The analysis of different CD4^+^ T cells (IL‐4, IL‐17, and IFN‐γ). Data are shown as mean ± SD. Differences with *P* < 0.05 (*), *P* < 0.01 (**), or *P* < 0.001 (***) were considered statistically significant, and ns means no significance.

As the vaccinia virus (VACA) belongs to the orthopoxvirus group and has been historically utilized in smallpox eradication efforts, it serves as an ideal attenuated model virus. To effectively demonstrate the virus‐neutralizing capabilities of AM@AEvs‐PB, mice were immunized three times with Evs, A29L, B6R, M1R, AMB, and AM@AEvs‐PB. Subsequently, the mice were challenged with VACA to assess their protective effects, assessing parameters such as weights, viral titers, and immune responses (**Figure** **6**A). Serum samples were collected from immunized mice and evaluated using a 50% microneutralization test (MRNT_50_) to determine neutralized capability. As shown in Figure 6B, mice immunized with AM@AEvs‐PB developed higher neutralizing titers than those in individual antigen groups, which were similar to those of mice in the AMB group. After i.m. administration, mice from all the groups were infected with VACA after 6 weeks. The infected mice were evaluated for weight loss and survival. Compared to the PBS group, mice immunized with individual antigens exhibited slight weight loss after the challenge, whereas those in the AMB and AM@AEvs‐PB groups exhibited nearly no weight loss (Figure 6C), demonstrating the better effective neutralization capability of multi‐antigen to the virus. No mortality was observed in any of the infected groups due to attenuated VACA (Figure S35, Supporting Information).

Additionally, in order to determine the viral load of mice quantitatively, when the mice were sacrificed on day 3 post‐challenge, we measured viral abundance by qPCR on samples from blood and lung according to the standard curve (Figure S36, Supporting Information). In the PBS group, viral RNA was detected at high average abundance levels in the blood (4.1 log_10_ copies per mL) using qPCR. However, in all vaccinated mice, the viral abundance was significantly reduced, with less than 2 log_10_ copies per mL on average (Figure 6D). Similarly, pulmonary viral RNA levels decreased from 6.2 log_10_ copies per mL in the PBS group to 5.5, 5.3, and 5.3 log_10_ copies per mL in mice vaccinated with A29L, B6R, and M1R, respectively. In contrast, viral RNA copies were decreased in both the AMB and AM@AEvs‐PB groups, with average reductions of 2.7 and 3.3 log_10_ copies in the lung compared to the PBS group (Figure 6E). In short, our bionic vaccine performs superior virus neutralization than both single antigens and AMB.

H&E staining was used to assess pulmonary lesions, and the results revealed severe notable lung lesions in mice treated with PBS, characterized by inflammatory infiltrates and thickening of the alveolar walls. Conversely, the AM@AEvs‐PB vaccination group exhibited elevated visibility of the pulmonary alveolus and a significant reduction in polymorphonuclear neutrophils, while the mice immunized with either A29L, B6R, or M1R still displayed slight alveolar wall thickening (Figure 6F), which announces the higher effort of AM@AEvs‐PB in prevent virus than those in single antigens. Interestingly, no significant changes could be observed in other major organs, including the heart, liver, spleen, and kidney among all the groups, as compared to the control group (Figure S37, Supporting Information). These results demonstrate that our synthesized vaccine displays substantial neutralization of VACA and behaves potent protective effects against MPV.

Moreover, we delved into its ability to provoke immune memory. As shown in Figure 6G, the subpopulations of central memory (CD44^+^ and CD62L^+^) and effector memory (CD44^+^ and CD62L^−^) T cells in CD4^+^ T cells were significantly promoted in AM@AEvs‐PB immunized mice compared to mice in the PBS group. The number of memory T cells in the bionic vaccine group exceeded that in the AMB group, suggesting that AM@AEvs‐PB considerably enhanced the T‐cell immune memory response due to the presence of AEvs. In addition, plasma cells in the lymph node remained at levels similar to those observed pre‐challenge in the bionic vaccine group, indicating the persistence of antibody titers (Figure 6H). The prolonged bionic nanovaccine enables playing a role of self‐adjuvating, and thus obviously increasing innate immunity by motivating the APCs, orchestrating the adaptive immunity through eliciting neutralizing antibodies of MPV.

Besides, the splenic CD4^+^ T cells were further investigated after challenge. As illustrated in Figure 6I, Figures S39–S41 (Supporting Information), the presence of the virus could increase IL 4^+^ CD4^+^ T‐cell numbers compared to the control group. Immunized mice exhibited a larger number of IL 4^+^ CD4^+^ T cells, especially in the AM@AEvs‐PB group, with a 1.5‐fold increase in the PBS group. Similarly, the number of IFN‐γ‐secreting CD4^+^ T cells in our synthesized vaccine group was larger than in other groups. However, while the proportion of IL‐17‐secreting CD4^+^ T cells in the PBS group was substantial at 0.81%, this proportion decreased in vaccinated mice. Briefly, the inflammation caused by the virus was alleviated by the enhanced anti‐virus response, owing to the immune memory responses evoked by the vaccine. In conclusion, our constructed bionic vaccine could effectively protect against the virus through immune memory and reducing viral inflammation during challenge.

Besides, in order to evaluate the potential of bionic vaccine system to become a platform for other infectious diseases, we preliminary explored the immunogenicity assessment of the RBD‐coated bionic vaccines. Compared with single RBD‐treated groups, bionic vaccines‐bearing groups possessed the enhanced specific titer (Figure S42, Supporting Information). Combined with the above results, the pre‐activated macrophage‐derived vesicles‐based system is a promising platform for the prevention and control of infectious diseases.

## 3 Discussion

MPV has emerged as a significant threat to global public health since its outbreak in July 2022. Though certain medications, such as Cidofovir, Tecovirimat, and tecovirimat, have received emergency authorization from the Centers for Disease Control and Prevention for MPV treatment, the lack of MPV‐specific medicine underscore the pressing need to develop an accessible vaccine as the most promising strategy for preventing MPV transmission. Live‐attenuated vaccines (such as JYNNEOS and ACAM2000) is a typical vaccine, but the limited specificity hinders its development in defending Mpox; mRNA vaccines, the next‐generation vaccination strategies, persist challenges pertaining to the efficient delivery of mRNA within the body and stringent transportation conditions because of their instability. Protein subunit vaccines contain viral or pathogenic proteins or polysaccharides, offering effective immunity protection without causing obvious negative effect, which present competitive advantages in terms of improved safety, stability, and immunogenicity.

Selecting immunogenic candidates for preventing MPV needs to be considered preferentially. EEV and IMV are two distinct infectious particles during its infection and replication cycles: EEV is responsible for intercellular transport and binds to host cell membranes, while IMV is more stable and affects the host‐to‐host transport, which made it suitable to construct recombinant protein vaccines with EEV and IMV antigens. A29L, M1R, and B6R are typical IMV and EEV antigens separately, which have been reported the effective inhibition toward the transport of MPV, and the combination of IMV‐ and EEV‐specific immunogens has been found to provide more protection than either immunogen alone. The lack of immunogenicity and easy enzymatic degradation are still the major shortages of the protein subunit vaccine. The engineered vesicles derived from immune cells can effectively address these issues: On the one hand, the vesicles act as antigens carrier via protecting cargos from enzymatic degradation. on the other hand, as the natural adjuvant, the preserved specific receptors on the vesicles can interacted with the APC, which enhanced and accelerated the antigen presentation. Besides, comparing to the general carrier liposome, vesicles exhibit high biocompatibility, obvious cell‐specific affinity, and minimal immune background interference. Our self‐adjuvant bionic vaccine, AM@AEvs‐PB, encapsulating EEV antigens (A29L and M1R) with the IMV antigen (B6R)‐decorated pre‐activated macrophage‐derived vesicles has been systematically constructed for Mpox prevention and achieves the initiation of innate immune and MPV‐specific adaptive immune.

We first verify the feasibility of the macrophage‐pre‐activating strategy. iNOS, the typical marker of activated macrophages, displayed increased expression as the integration of antigens, which demonstrates the successful stimulation of macrophages. During the process, the activation of Toll‐like receptor signaling and inflammatory factor receptor pathways (cytokine and cytokine receptors, TNF signaling, and chemokine signaling), which are implicated in viral defense and antiviral response, has been validated via further RNA sequencing result analysis. Furthermore, we confirm that the crucial role of NLRP3 inflammasome activation in antigens‐stimulated macrophage during the pre‐activation process according to the upregulation of caspase‐1, NLRP3, and NF‐κB protein expression. Besides, the NLPR3 pathway related biomarkers IL 6 and TNF α also significant increase in antigens (A29L, B6R, and M1R) groups. In short, combining the results of RNA‐seq, the antigens activate the macrophages depending on NLRP3 inflammasome pathway, and signify the ability of the MPV antigen to elicit macrophage‐mediated innate immunity against the virus. In order to fabricate a bionic vaccine, the pre‐activated macrophages were collected, lysed, and reconstructed to form activated vesicles (AEvs), and then load with IMV antigens A29L and M1R, following the conjugation with PEGylated B6R to form the system AM@AEvs‐PB. The characterization of AM@AEvs‐PB confirms that particles were effectively fabricated, with a size of 100 nm and surface charge of ≈ ‐20 mV. Importantly, AM@AEvs‐PB exhibits superior biocompatibility and systemic safety both in vitro and in vivo.

AM@AEvs‐PB needs to be recognized and internalized by matured APCs to effectively trigger immune responses. Compared to the antigen alone, AM@AEvs‐PB exhibits more robust cellular uptake and prolonged retention via the alternative COVA@AEVs model due to the lack of fluorescence‐labeled MPV proteins. The activation and maturation of APCs by AM@AEvs‐PB is crucial for initiating innate immune responses. Our data demonstrates that pre‐activated macrophage‐derived vesicles synergistically enhance the maturation of BMDMs and BMDCs in the AM@AEvs‐PB group. This enhancement leads to heightened antigen immunogenicity, bolstered innate immune response, and reinforced cytokine induction, acting in a manner similar to that of an adjuvant. Interestingly, vesicles derived from inactive macrophages lack these adjuvant‐like effects. As reported previously, self‐DNA damage and tumor DNA are the three key factors causing the activation of cGAS‐STING signaling,^[^ ^22^ ^]^ and monkeypox is a typical kind of DNA poxviruses, which cause human and veterinary diseases. After exploration, we found the vesicles‐based vaccine triggered the upregulation of cGAS and STING protein expression in both BMDM and BMDC cells compared to the control group, suggesting the activation of cGAS‐STING pathway by the vesicles. This suggests that the acquisition of adjuvant functionality by vesicles depends on macrophage activation and the subsequent expression of the corresponding receptors.

Additionally, the adaptive immune responses in vivo reveal that the mice in the AM@AEvs‐PB group exhibit elevated titers of specific antibodies, augmented proportions of cellular immune markers, and enhanced response in GC B cells compared to the control and antigen‐only groups. Subsequent analysis of cellular immune markers reveals increased proportions of IL‐4, IL‐17, and IFN‐γ‐producing CD4^+^ T cells in the AM@AEvs‐PB group. IL‐4, IFN‐γ, and IL‐17 are associated with humoral immune responses, antiviral defenses, and inflammatory reactions, respectively. The increased number of IFN‐γ‐secreting and IL‐4‐secreting CD4^+^ T cell, which represented the Th1 cells and Th2 cells separately. Besides, we determined the cellular immunity and humoral immunity by measuring CD8^+^ T cells and GC B cells, respectively. Obviously, the proportion of CD8^+^ T cells and the ratio of GC B cells elevated in the bionic vaccine compared to the other groups, which suggested the bionic vaccine induced an antigen‐specific CD4^+^ T cell response biased toward mixed Th1/Th2 phenotype. Furthermore, Mpox infection elicits a prominent mixed Th1/Th2 immune response with elevated levels of Th2‐associated cytokines (IL‐4, IL‐5, IL‐6, IL‐10) and Th1‐associated cytokines (IL‐2, IL‐12, TNF‐α, IFN‐γ).^[^ ^24^ ^]^ These augmented T‐cell subpopulations suggest a mild inflammatory response post‐immunization in mice, strengthening both humoral and cellular immune defenses against viral pathogens. This comprehensive immune response substantially contributes to an effective viral defense.

In the subsequent challenges with VACA, our constructed bionic vaccine demonstrates the ability to prevent viral infection‐related side effects, including weight loss and pulmonary fibrosis. Additionally, it significantly reduces viral titers in both the lung and blood following intranasal administration. Besides, vaccinated mice exhibit decreased proportions of IL‐17‐producing CD4^+^ T cells, indicating reduced inflammation. Conversely, proportions of IL‐4‐ and IFN‐γ‐producing CD4^+^ T cells are increased, suggesting heightened cellular and humoral immune responses. Importantly, the bionic vaccine promotes sustained memory responses, explaining how a robust adaptive immune response alleviated inflammation. These outcomes highlight the potential of the biomimetic vaccine to provide lasting protection against MPV.

Compared to other type of vaccines, our programmable macrophage vesicle‐based bionic vaccine performs outstanding specificity with few side effects. Distinct from live‐attenuated vaccines, it poses no biological safety risks as it comprises approved antigens, not pathogens. Relative to mRNA vaccines, our vesicle vaccine demonstrates remarkable stability in PBS and FBS, and is amenable to lyophilization and room‐temperature storage. Unlike mRNA, which requires carrier assistance for cellular uptake, our vesicles offer optimal preservation and biodistribution. Despite these advantages, transitioning our vaccine from pre‐clinical to clinical trials presents challenges. Biological safety, confirmed preliminarily in vitro and in vivo, must be further assessed in diverse animal models, including humanized mice and non‐human primates, to ensure safety and efficacy. After the animal model tests, the bionic vaccines also need a during and repetitive verification in diverse populations to affirm the reliability of clinical trial. Additionally, rigorous quality control is essential, particularly during vesicle harvest, as these vesicles are crucial for vaccine formulation.

In summary, for MPV prevention, we fabricated a bionic self‐adjuvant vaccine AM@AEvs‐PB based on MPV antigens pre‐activated macrophage‐derived vesicles, loading with the IMV protein (A29L and M1R), combining with EEV antigen (B6R) superficially to effectively counter MPV replication cycles. The pre‐activated macrophages‐derived vesicles promote the delivery of antigens and prolong the retention time. Besides, the activated vesicles and MPV antigens synergistically motivated innate immunity, and further orchestrate the adaptive immunity through eliciting neutralizing antibodies, enhancing cellular immunity, and elevating B cell responses and memory effect. Though suboptimal in vivo persistence and the need for enduring protective efficacy remain focal concerns for the vaccine, the introduction of long‐term carriers like hydrogel and microneedles can overcome these shortcomings. Besides, expanded animal studies, encompassing humanized mice and non‐human primates, should be conducted to comprehensively ascertain the vaccine's efficacy in the future. In all, our constructed programmable macrophage vesicle simulating bionic self‐adjuvanting vaccine holds the potential to provide a valid tool for immunizing against monkeypox virus.

## 4 Experimental Section

### Materials

N‐hydroxysuccinimide (NHS), 1‐(3‐dimethylaminopropyl)‐3‐ethylcarbodiimide hydrochloride (EDC), and BSA were purchased from Sigma (USA). DSPE‐PEG_2000_‐COOH was obtained from Ruixibio Technology Co., Ltd (Xi'an, China). The Cy5‐labeled OVA was purchased from Bioss (Beijing, China). The cell lines RAW 264.7, DC 2.4 and HUVEC were purchased from ATCC (USA). The female Balb/c mice (6 weeks old) and C57BL/6 mice (6 weeks old) were purchased in Ruige company (Guangzhou, China), and All animal experiments were approved by the Animal Welfare Committee and conducted in accordance with the regulations of the Guangdong Medical Laboratory Animal Center (No. D202310‐1).

### Collection and Purification of MPV Antigens

The MPV antigens A29L, B6R, and M1R were expressed and purified by *E. coli* expression system. The sequence of each protein was downloaded from the gene bank (Genebank No. YP_010377135.1, URK20605.1, and URK20517.1 for A29L, B6R, and M1R, separately) and constructed in *PET‐28a* vector, following selected the single colonies and expanded. The amplified cells were lysed and purified with his label protein agarose. The purified antigens were placed in a dialysis bag (3 kDa) and dialyzed at 4 °C within PBS for 24 h, and then concentrated to 1 mg mL^−1^ and stored at −80 °C.

### Preparation of AM@AEvs‐PB

As displayed in Figure S43 (Supporting Information), RAW 264.7 cells were seeded in plates and then cultured with 2 µg MPV antigen A29L, B6R, and M1R to obtain the preactivated macrophage. The activated macrophages were collected, lysed with hypotonic lysate, gradient centrifugated (first 3000 g, 5 min, and then 12 000 rpm, 30 min), and then extruded through polycarbonate membranes with pore sizes of 800 nm, 400 nm, and 200 nm to acquire activated vesicles (AEvs). 1 mL AEvs (1 mg mL^−1^) was mixed with IMV antigens A29L and M1R to form AM@AEvs. Meanwhile, the EEV antigen of MPV B6R was PEGylated using DSPE‐PEG_2000_‐COOH through dehydration and condensation under 4 °C overnight with the addition of NHS and EDC (molecular ratio = 1:1), and finally formed PEGylated B6R (PEG‐B6R). The PEG‐B6R was added into the system AM@Evs, followed by ultrafiltration trice using an ultrafiltration tube with cut‐off size of 10 kDa, and the AM@AEvs‐PB was obtained after removing the unligated DSPE‐PEG_2000_‐COOH in the lower tube.

### BMDMs and BMDCs Isolation and Culture

BMDMs and BMDCs were derived using standard protocols. In brief, bone marrow cells were isolated from C57BL/6 mice (for BMDM) or Balb/c mice (for BMDC), and cultured in culture medium with 10% heat‐inactivated FBS (Gibco) and 1% penicillin–streptomycin. The addition of 10 ng mL^−1^ M‐CSF (Peprotech) for BMDMs in DMEM (Gibco), and 10 ng mL^−1^ IL‐4 (Peprotech), and 20 ng mL^−1^ GM‐CSF (Peprotech) for BMDCs in RMPI 1640 (Gibco). BMDM and BMDCs could be collected and used for experiments after 7 days.

### In Vitro Antigen Processing

The activation and maturation were studied by detecting the upregulation of markers on the APC. BMDMs and BMDCs were cultured with Evs, AEvs, A29L, B6R, M1R, AMB, AMB@Evs, AM@AEvs‐PB, while PBS and LPS were set as negative and positive control, respectively. After 24‐hour incubation, the cells were analyzed using a Flow cytometer (Beckman Coulter). Before the tests, the BMDMs were first blocked and then were cultured with APC anti‐mouse CD11b (eBioscience), FITC anti‐mouse F4/80 (eBioscience), and PE anti‐mouse CD86 (eBioscience) antibodies; the isolated BMDCs incubated with FITC anti‐mouse CD11c (eBioscience), Percp‐Cy5.5 anti‐mouse MHC‐II (Elabscience), APC anti‐mouse CD80 (eBioscience) and PE anti‐mouse CD86 antibodies (eBioscience). All antibodies were diluted in the staining buffer according to the manufacturer's suggestions. Cells were gated on the basis of fluorescence‐minus‐one controls, and the frequencies of cells staining positive for each marker were recorded.

### Detection of Cytokine Secretion of BMDMs and BMDCs

After the BMDMs and BMDCs were separated into 10 groups (control, Evs, AEvs, A29L, B6R, M1R, AMB, AMB@Evs, AM@AEvs‐PB, and LPS) by different stimulation and activation, the supernatants of cells were collected after 24‐hour co‐culture and then evaluation using ELISA kits (Dakewe Biotech, China). BMDMs secreted TNF‐α and IL‐6, BMDCs secreted IFN‐γ were tested in the detection.

### In Vitro Internalization and In Vivo Retention Tests

The COVA was first loaded into the AEvs to form COVA@AEvs according to the protocol of AM@AEvs‐PB. Immunofluorescence staining was used to track the in vitro internalization. RAW 264.7 cells and DC 2.4 cells were co‐cultured with COVA and COVA@AEvs for different time intervals (0–8 h). Nuclei and F‐actin were labeled with Hoechst 33342 (blue) and Phalloidin (red) after fixation and permeabilization, and images were captured by CLSM (A1, Nikon, Japan). The experimental procedures were approved by the Institute of Pharmacology and Toxicology Academy of Military Medical Sciences PLA, Peop. Rep. China Ethical Committee on Animal Care and Use, and all efforts were made to minimize animal suffering and reduce the number of animals used for the experiments.

### In Vivo Retention Tests

For in vivo retention, equivalent doses (0.5 mg kg^−1^) COVA in COVA@AEvs and COVA were prepared and administrated intramuscularly into the Balb/c nude mice after isoflurane anesthesia, following tracing the retention via AniView 600z (BLT, China) during different period of time (from 0 to 48 h).

### In Vivo Titer Test

6‐week‐old female Blab/c mice (*n* = 5) were immunized with PBS, A29L, B6R, M1R and AM@AEvs‐PB for three times. The immune dose of antigen is 2 µg per mouse every time, and mice in A29L, B6R, and M1R groups were immunized antigen with aluminum salts adjuvant (Bioss, China, 50 µg per mouse every time). The blood was taken every 2 weeks, and employed to the titer test.

For the titer test, microtiter plates (Nunc Cell Culture, Thermo Fisher) were coated with 10 µg mL^−1^ RBD in 100µl coating buffer (R&D Systems) and incubated overnight at 4 °C. Wells were then blocked with 1% (w/v) bovine serum albumin (BSA; Sigma‐Aldrich) for 1 h at 37 °C. After washing three times with PBS‐T, serial dilutions (1:100, 1:1,000, 1:5,000, 1:10,000, 1:50,000, 1:100,000, 1:200,000) of sera samples were added, and control sera samples with 1:100 dilution were added into wells for incubation for 1.5 h at 37 °C. Then, samples were washed with PBS‐T three times and then incubated with HRP‐labelled anti‐mouse IgG secondary antibody (Sangon) at a 1/2,000 dilution (100 µL per well) at a 1/20,000 dilution (100 µL per well) for 1 h at 37 °C. After washing four times with PBS‐T, 3,3’,5,5’‐tetramethylbenzidine soluble substrate (TMB; Thermo Fisher) was added to each well (100 µL per well). After incubation of 30 min at room temperature, 50 µL of stop solution was added and optical absorption at 450 nm was determined by a plate reader. The end‐point titre of IgG was quantified by the reciprocal of maximal serum dilution that exceeded twice the s.d. above the mean readout of the control group. The individual antibody titres are shown as [log10(−x ± s.d.)], calculated as the reciprocal of maximal serum dilution.

### In Vivo Antigen Presentation, Cellular Response, Humous Response, and Immune Memory Evaluation

6‐week‐old female Blab/c mice (*n* = 3) were immunized with PBS, A29L, B6R, M1R and AM@AEvs‐PB for three times. The immune dose of antigen is 2 µg per mouse every time, and mice in A29L, B6R, and M1R groups were immunized antigen with aluminum salts adjuvant (Bioss, China, 50 µg per mouse every time). The spleen and lymph nodes from immunized mice were collected 2 weeks after the last immunization for flow cytometric analysis. Cell suspensions from spleen and lymph node were prepared by abrading and pressing of the tissue through 40 µm cell strainers, and the splenic cells were further treated with the erythrocyte lysate (Solarbio, China) before the examination by flow cytometry while the lymph cells could be tested without pre‐treating lysate. The isolated cells were first blocked with 5% BSA, and then incubated with FITC anti‐mouse CD11c (eBioscience), APC anti‐mouse CD80 (eBioscience), PE anti‐mouse CD86 (eBioscience) and Percp‐Cy5.5 anti‐mouse MHC‐II (Elabscience) antibodies for DC detection. For T cells, the CD4^+^ T cells were incubated with FITC anti‐mouse CD45 (Biolegend), PerCP‐Cy5.5 anti‐mouse CD3e (Biolegend), BV510 anti‐mouse CD4 (Biolegend), PE anti‐mouse IL‐4 (Biolegend), Alexa Fluor 700 anti‐mouse IFN‐γ (Biolegend) and Alexa Fluor 647 anti‐mouse IL‐17 antibodies (Biolegend), and CD8^+^ T cells were cultured with FITC anti‐mouse CD45 (Biolegend), PerCP‐Cy5.5 anti‐mouse CD3e (Biolegend), APC anti‐mouse CD8a (Biolegend), anti‐mouse BV421 granzyme B (Biolegend) and PE anti‐mouse perforin (Biolegend) antibodies. As for GC B cells in lymph node, the cells were stained with Percp‐Cy5.5 anti‐mouse CD19 (Elabscience), BV421 anti‐mouse B220 (BioLegend), APC anti‐mouse CD95 (Fas) (Biolegend) and PE anti‐mouse GL‐7 (eBioscience) antibodies. For immune memory, APC anti‐mouse CD62L (Elabscience) and PE anti‐mouse CD44 (Elabscience) were co‐cultured with the CD4^+^ T cells. All the antibodies were diluted in staining buffer with the recommended ratio, and the frequencies of cells staining positive for each marker were recorded, and the mean values of the positive cells were calculated with 3 mice.

### Protection Efficacy Evaluation

Briefly, 6‐week‐old Balb/c mice (eight mice each group) were challenged with 10^5^ PFU of VACA 14 days after three times vaccination with Evs, A29L, B6R, M1R, AMB, or AM@AEvs‐PB in 6 weeks. The blood was obtained before the challenge for the viral neutralization. The weight loss and survival rate were monitored for 3 days post challenge, and the mice were sacrificed at day 4, with detecting the viral RNA copies in blood and lungs by qPCR (Quantstudio 1, ThermoFisher, USA) using the viral DNA/RNA kit (Vazyme, China), and determining the humous immune and cellular immune by flow cytometry.

### Statistical Analysis

All experiments were carried out in triplicate, and the results are expressed as the mean ± SD. Statistical analysis was performed using GraphPad Prism 8. The two‐tailed Student's t‐test was utilized to analyze the difference between two groups, and the difference in three or more groups was analyzed by one‐way ANOVA for multiple comparisons. Differences with *P* < 0.05 (*), *P* < 0.01 (**), or *P* < 0.001 (***) were considered statistically significant.

## Conflict of Interest

The authors declare no conflict of interest.

## Author Contributions

W.Q.L., C.G.S., M.J.L., and S.C.M. contributed equally to this work. J.D.Z., Q.M., C.G.S., Y.D.J. performed conceptualization. W.Q.L., M.J.L., J.L.H., Z.N.R. performed methodology. S.C.M., C.X.L., S.G., K.Y.W. performed software. M.H.Z., Q.L.X., Y.C.Y. performed visualization. J.D.Z., C.G.S., Y.H.L., Y.D.J., W.W. performed supervision. W.Q.L., J.L.H. performed writing—original draft. J.D.Z., C.G.S., and Y.H.L. performed writing—review & editing.

## Supporting information



Supporting Information

## Data Availability

RNA‐seq data have been deposited at GEO and are publicly available as of the date of publication. Accession number is listed in the key resources table. This paper does not report original code. Any additional information required to reanalyze the data reported in this paper is available from the lead contact upon request.
