# Correction to Programmable Macrophage Vesicle Based Bionic Self‐Adjuvanting Vaccine for Immunization Against Monkeypox Virus

**DOI:** 10.1002/advs.202509423

**Published:** 2025-06-19

Weiqiang Lin, Chenguang Shen, Mengjun Li, Shengchao Ma, Chenxin Liu, Jialin Huang, Zuning Ren, Yuechao Yang, Minghai Zhao, Qiulin Xie, Shuang Guo, Wei Wang, Kaiyuan Wang, Qiang Ma, Yideng Jiang, Judun Zheng, Yuhui Liao. Programmable Macrophage Vesicle Based Bionic Self‐Adjuvanting Vaccine for Immunization against Monkeypox Virus. Adv. Sci. 2025, 12, 2408608. https://doi.org/10.1002/advs.202408608.

In the Data of the original publication, the image representing the fluorescence images after I.m injection with COVA@Evs and COVA was incorrectly presented in Figure 3E. As the quantification analysis of the corresponding mice area (Figure 3E) was based on the correct images, the results and conclusions remain unchanged. The correct image should be as follows:


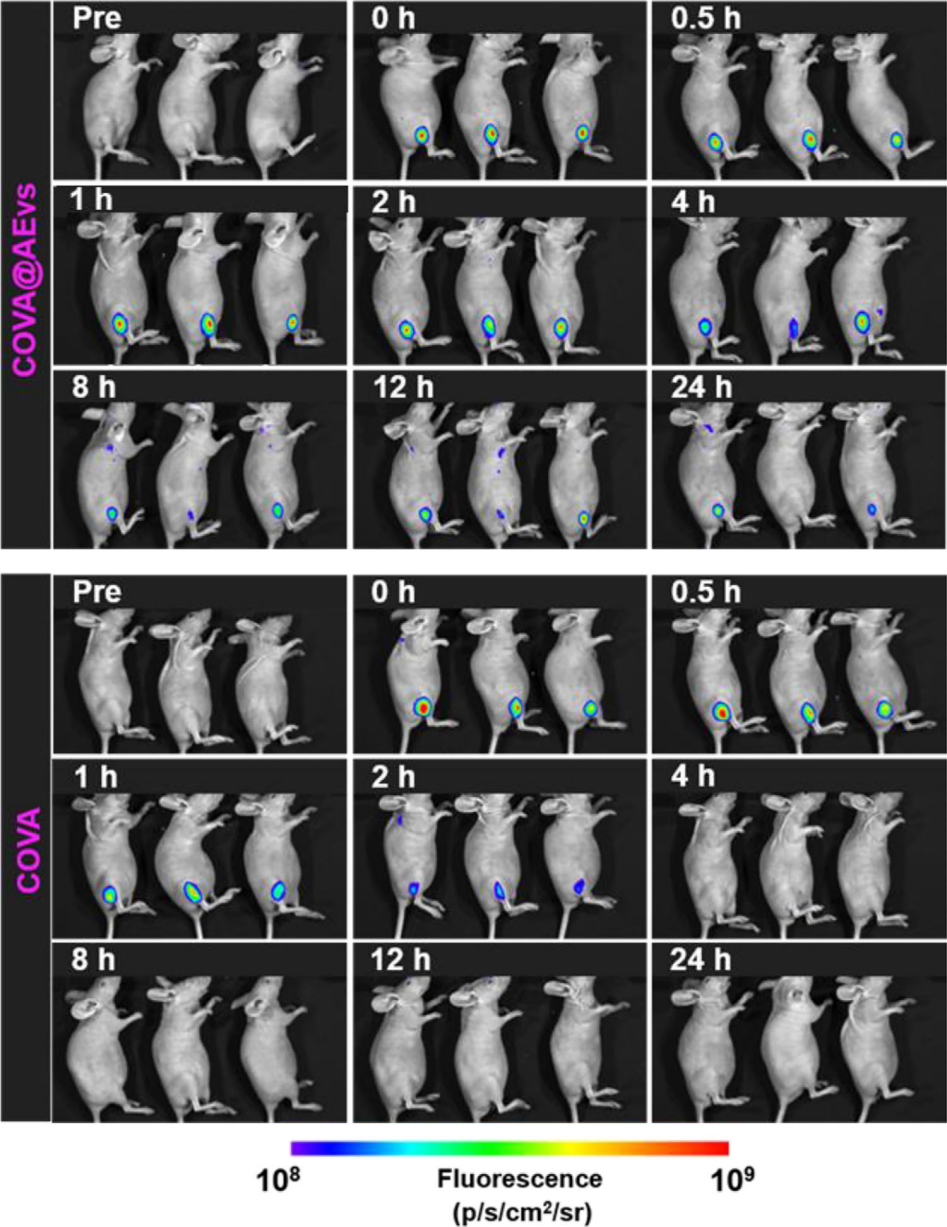


Figure 3E. The fluorescence images after I.m injection with COVA@Evs and COVA.

The image representing the subpopulation of antigen specific splenic IL‐17^+^ CD4^+^ T cells of vaccinated mice with PBS, AMB, and AM@AEvs‐PB 3 days after the challenge was incorrectly presented in Figure S40 (Supporting Information). As the quantification analysis of the corresponding flow results (Figure S40, Supporting Information) was based on the correct images, the results and conclusions remain unchanged.

The correct image should be as follows:


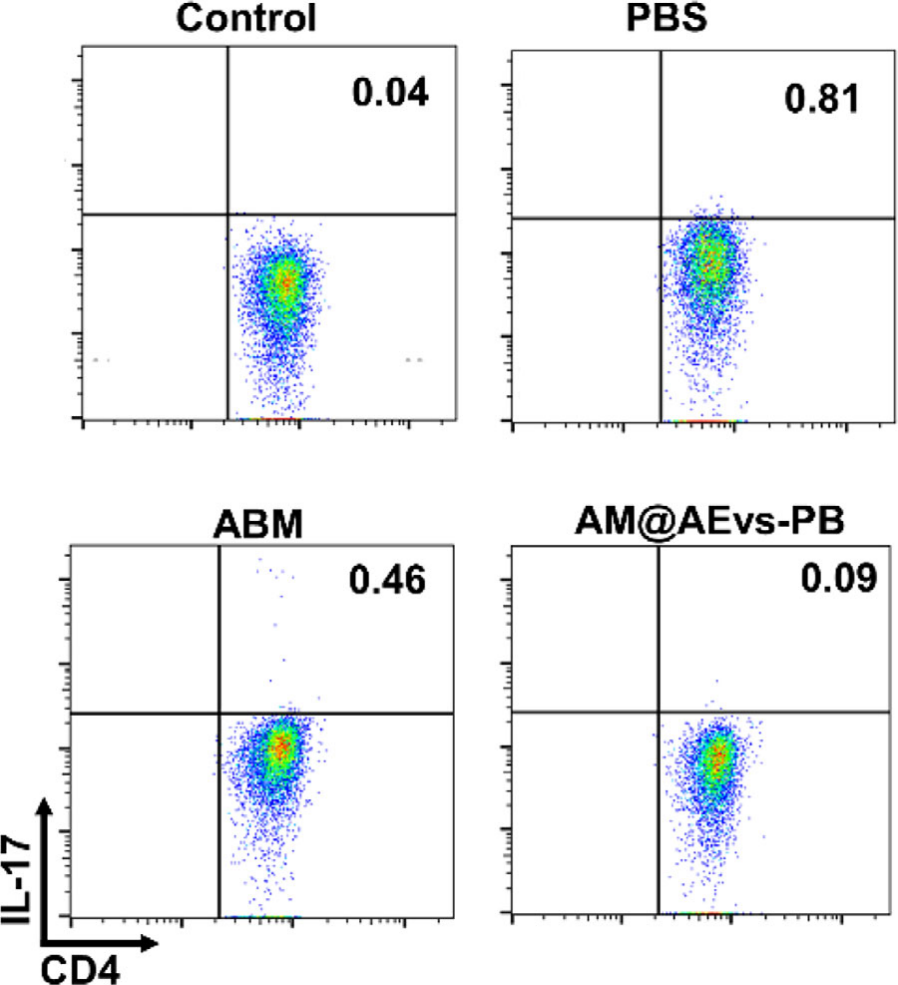


Figure S40. The subpopulation of antigen specific splenic IL‐17^+^ CD4^+^ T cells of vaccinated mice with PBS, AMB, and AM@AEvs‐PB 3 days after challenge.

We apologize for this error and for the inconvenience it may have caused.

