# Supplementary material for: Programmable Macrophage Vesicle Based Bionic Self‐Adjuvanting Vaccine for Immunization against Monkeypox Virus
Source: Adv Sci (Weinh). 2024 Nov 8;12(1):2408608. doi: 10.1002/advs.202408608 (PMC11714231; doi:10.1002/advs.202408608)
Supplement: Supplementary file 1 — Supporting Information [file ADVS-12-2408608-s001.docx]

# Programmable Macrophage Vesicle based Bionic Self-Adjuvanting Vaccine for Immunization against Monkeypox Virus

Weiqiang Lin^1,2‡^, Chenguang Shen^4‡^, Mengjun Li^4‡^, Shengchao Ma^1‡^, Chenxin Liu^5^, Jialin Huang^3^, Zuning Ren^4^, Yuechao Yang^3^, Minghai Zhao^3^, Qiulin Xie^3^, Shuang Guo^1^, Wei Wang^4^, Kaiyuan Wang^6^, Qiang Ma^5^*, Yideng Jiang^1*^, Judun Zheng^3^*, Yuhui Liao^1,2^*

1. NHC Key Laboratory of Metabolic Cardiovascular Diseases Research, Ningxia Key Laboratory of Vascular Injury and Repair Research, Ningxia Medical University, Yinchuan, 750004, P. R. China.
2. Institute for Engineering Medicine, Kunming Medical University, Kunming, 650500, P.

R. China.

1. Molecular Diagnosis and Treatment Center for Infectious Diseases, Dermatology Hospital, Southern Medical University, Guangzhou, 510091, P. R. China.
2. BSL-3 Laboratory (Guangdong), Guangdong Provincial Key Laboratory of Tropical Disease Research, School of Public Health, Southern Medical University, Guangzhou, 510515, P. R. China.
3. School of Laboratory Medicine and Biotechnology, Southern Medical University, Guangzhou, 510515, P. R. China.
4. Departments of Diagnostic Radiology, Surgery, Chemical and Biomolecular Engineering, and Biomedical Engineering, Yong Loo Lin School of Medicine and College of Design and Engineering, National University of Singapore, Singapore, 119074, Singapore.

*Corresponding Authors: [liaoyh8@mail.sysu.edu.cn](mailto:liaoyh8@mail.sysu.edu.cn) (Y. Liao); [zhengjd53815@163.com](mailto:zhengjd53815@163.com) (J. Zheng); [jydjwc@163.com](mailto:jydjwc@163.com) (Y. Jiang); [mq@smu.edu.cn](mailto:mq@smu.edu.cn) (Q. Ma).

# ‡These authors contributed equally to this work.

**Fig. S1.**


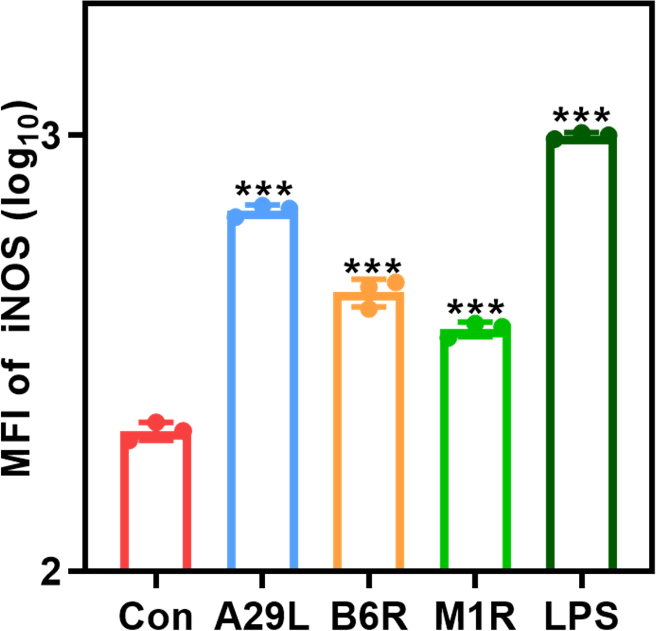


**Fig. S1.** Analysis of the iNOS expression in RAW 264,7 cells via flow cytometry.

# Fig. S2.

**150**


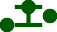

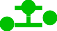

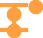

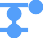

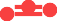


*******

***** *****

*******

**Relative Green Fluorescent Intensity (%)**

**100**

**50**

**0**

**Con A29L B6R M1R LPS**

**Fig. S2.** Analysis of relative fluorescence intensity of iNOS in immunofluorescence staining.

# Fig. S3.


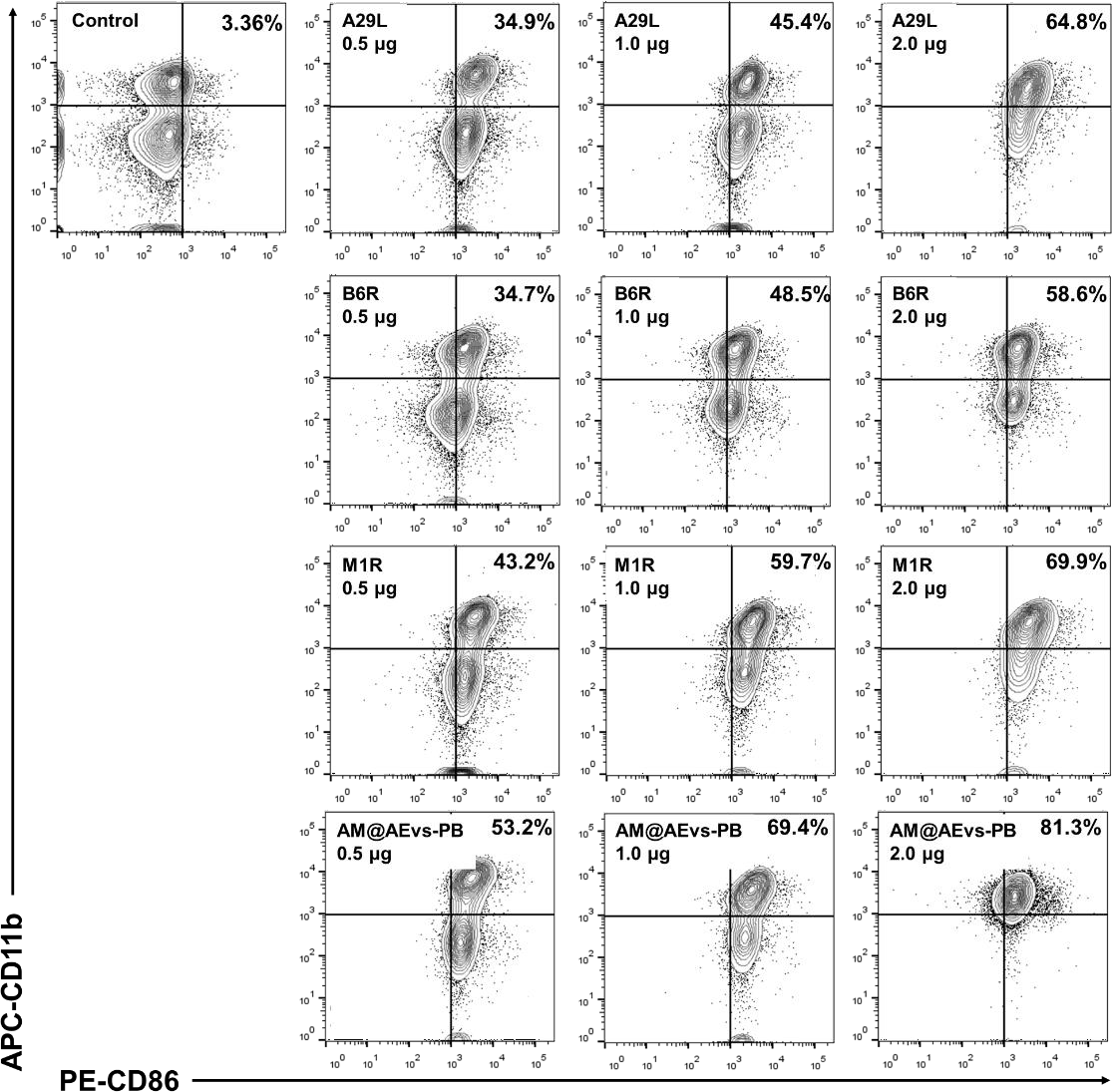


**Fig. S3.** The activation proportion of RAW 264.7 cells under different doses (0.5, 1.0 and

2.0 μg) of antigens (A29L, B6R and M1R).

# Fig. S4.


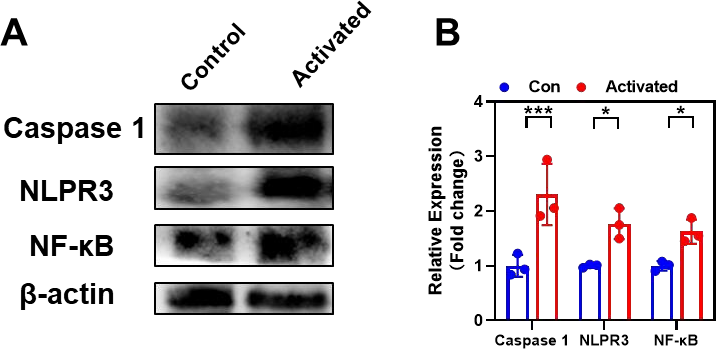


**Fig. R8. A)** Western blot analysis of the expression levels of STING pathway-related proteins in BMDM treated with AM@AEvs-PB for 24 h. **B)** Corresponding statistical analysis histograms of Caspase 1, NLPR3, and NF-κB (n = 3). Differences with P < 0.05 (*), P < 0.01 (**) or P < 0.001 (***) were considered statistically significant.

# Fig. S5.


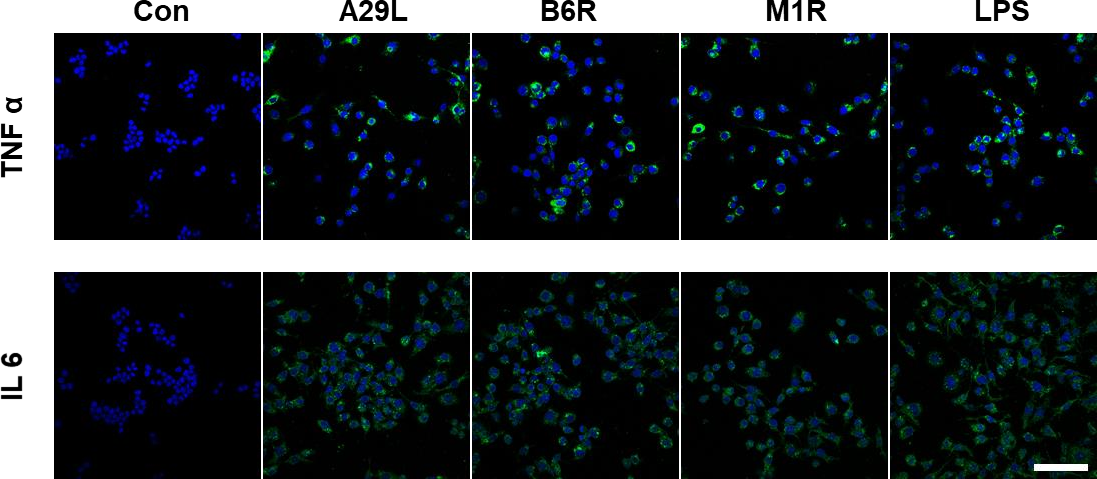


**Fig. S5.** Immunofluorescence staining (green fluorescence for TNF α and IL 6, and blue fluorescence for DAPI). Scale bar: 100 μm.

# Fig. S6.


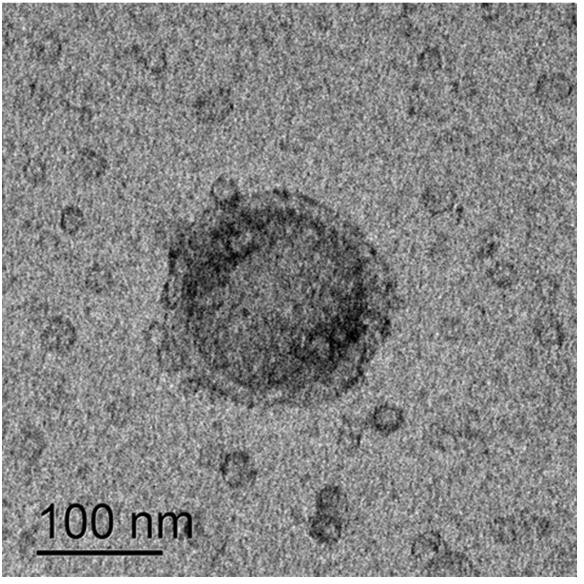


**Fig. S6.** The TEM image of AEvs.

# Fig. S7.


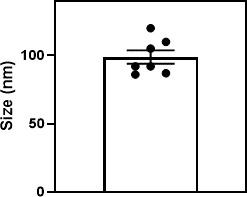


**Fig. S7.** Size statistic result of AM@AEvs-PB in TEM calculated by ImageJ software (n=7).

# Fig. S8.


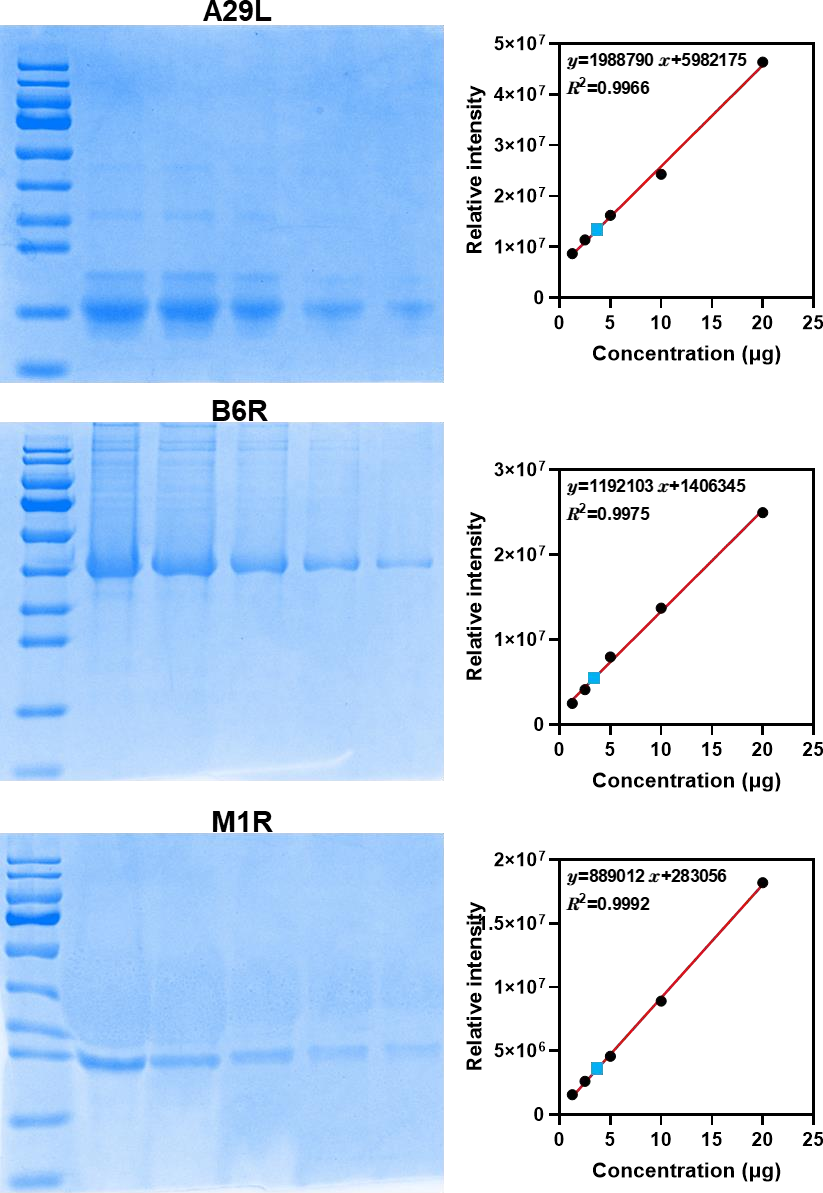


**Fig. S8.** Fig. R2. SDS-PAGE and the standard curve of A29L, B6R and M1R, and the doses are 20, 10, 5, 2.5, 1.25 μg from left to right (blue point: antigen concentration; black points: standard curve points).

# Fig. S9.

**150**


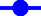


**FBS PBS**

**100**

**Size (nm)**

**50**

**0**

**0 2 4 6 8**

# Time (Days)

**Fig. S9.** Stability of AM@AEvs-PB in FBS and PBS for 7 days.

# Fig. S10.


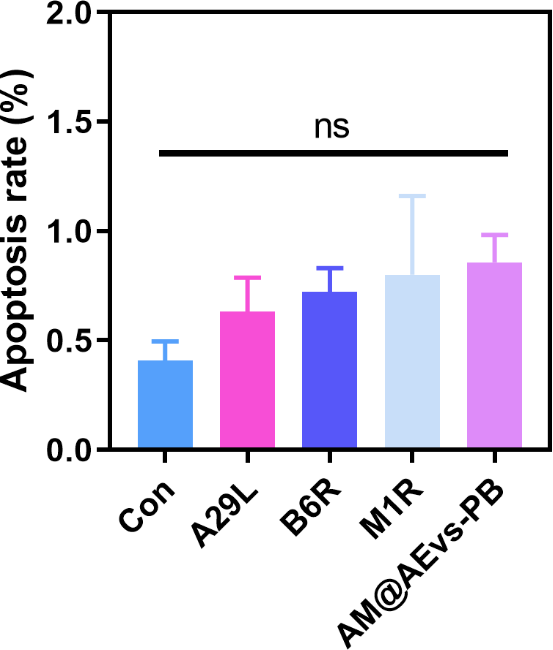


**Fig. S10.** Analysis of apotosis rate within 2 μg A29L, B6R, M1R and AM@AEvs-PB. (n=3; ns: no significance)

# Fig. S11.


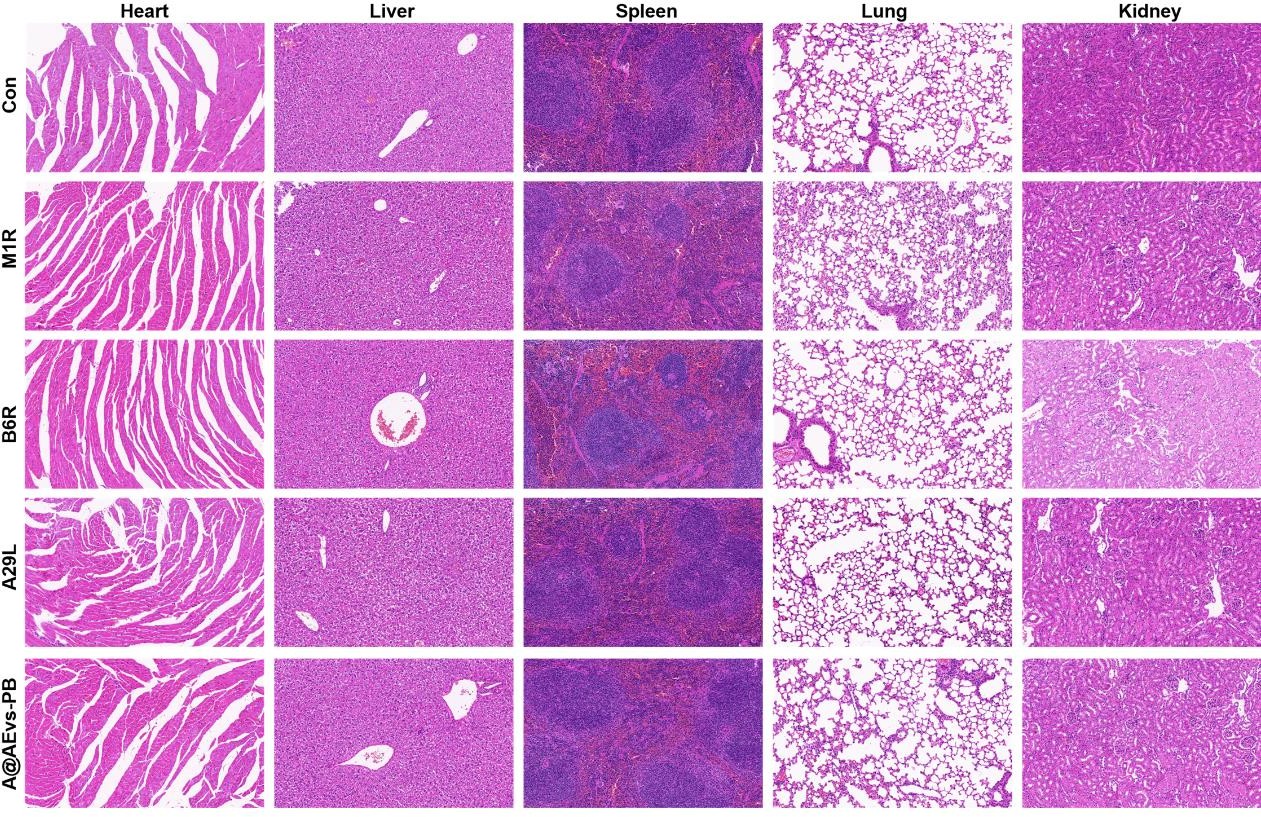


**Fig. S11.** H&E of the major organs (heart, liver, spleen, lung and kidney) within 2 μg A29L, B6R, M1R and AM@AEvs-PB via i.m.

# Fig. S12.


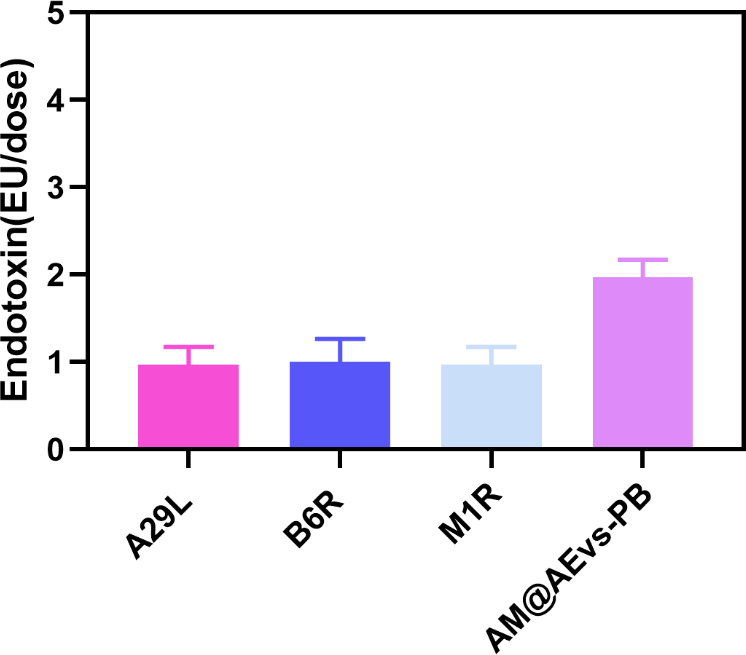


**Fig. S12.** Endotoxin levels of A29L, B6R, M1R and AM@AEvs-PB. The dose of mice was 2 µg.

# Fig. S13.


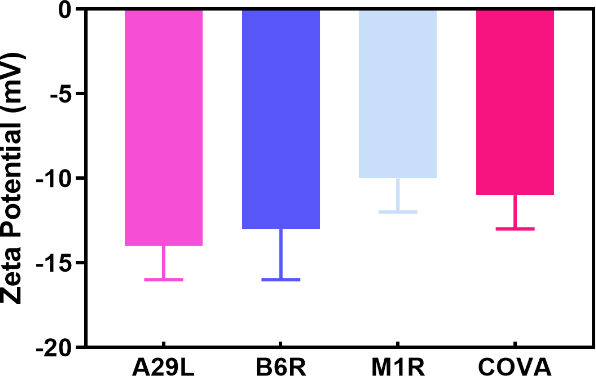


**Fig. S13.** The zeta potential of antigens (A29L, B6R and M1R) and COVA.

# Fig. S14.


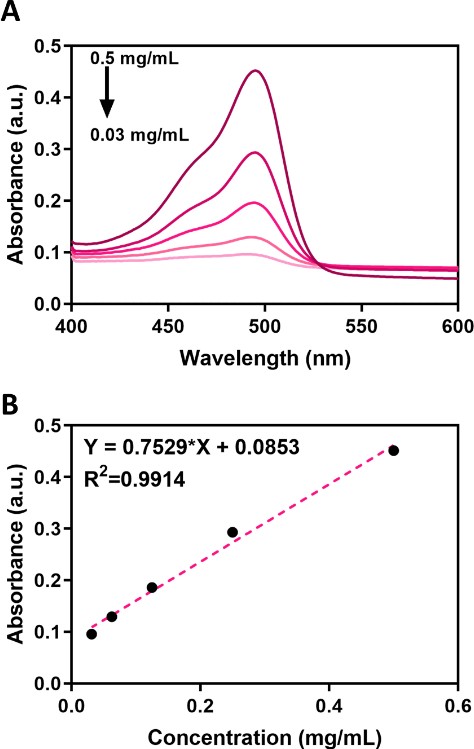


**Fig. S14. The standard curve of COVA. A)** Absorbance of COVA range from 0.03 mg/mL to 0.5 mg/mL *via* UV-vis spectra, and **B)** its standard absorbance curve.

# Fig. S15.


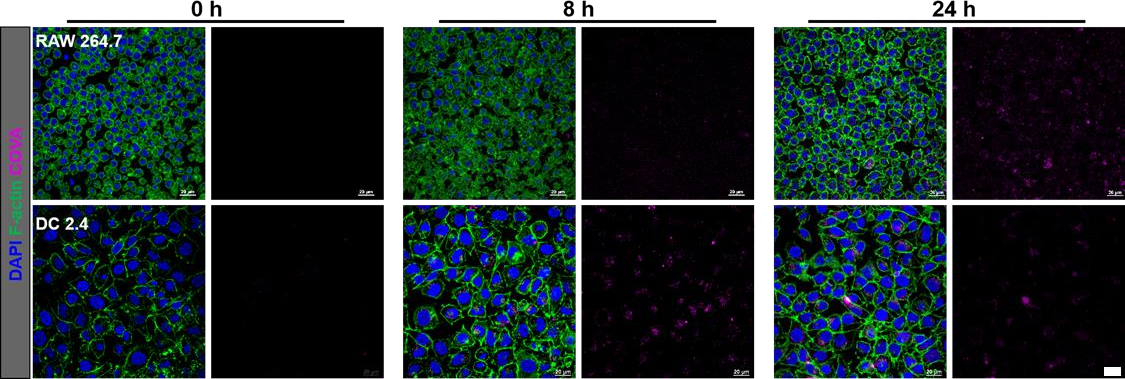


**Fig. S15.** The cellular uptake of COVA in RAW 264.7 cells and DC 2.4 cells during 24 h (Scale bar: 20 µm).

# Fig. S16.


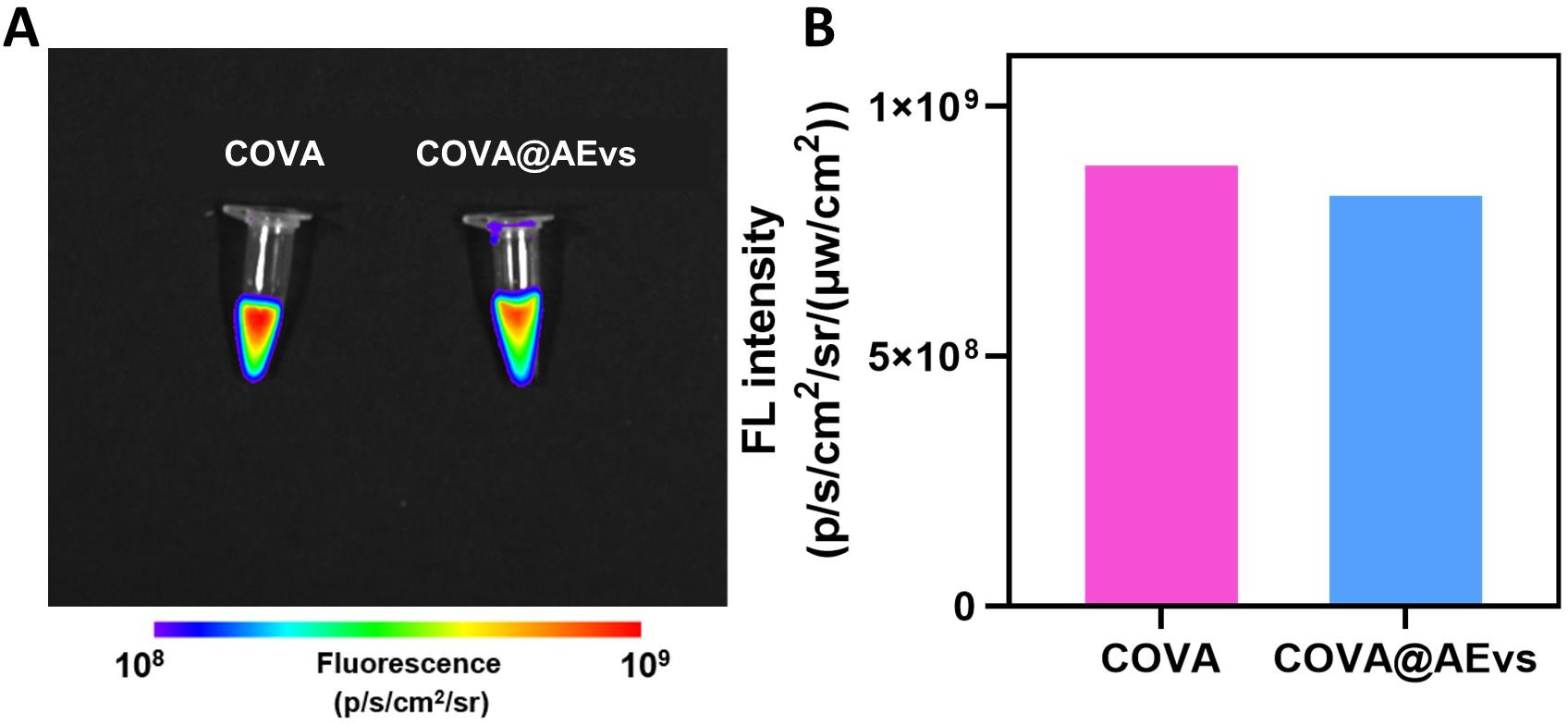


**Fig. S16.** The fluroescence images of COVA and COVA@AEvs.

#
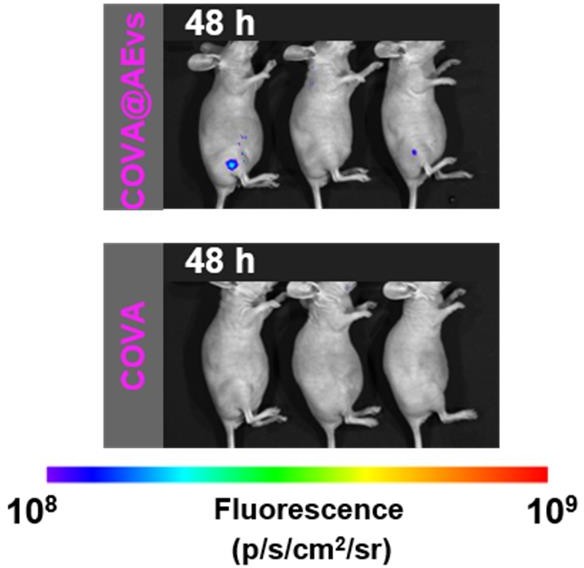
Fig. S17.

**Fig. S17.** The fluorescence images of mice post 48 h i.m. with COVA@AEvs and COVA.

# Fig. S18.


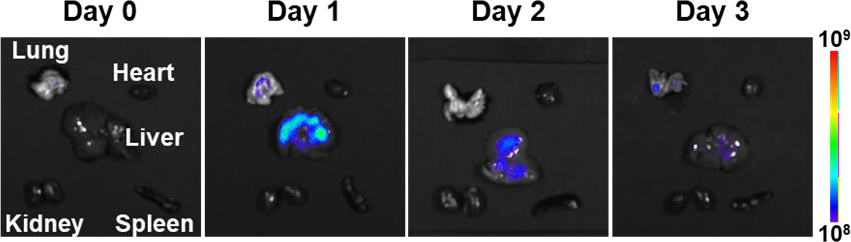


**Fig. S18.** Biodistribution of COVA@AEvs after intramuscular vaccinated.

# Fig. S19.


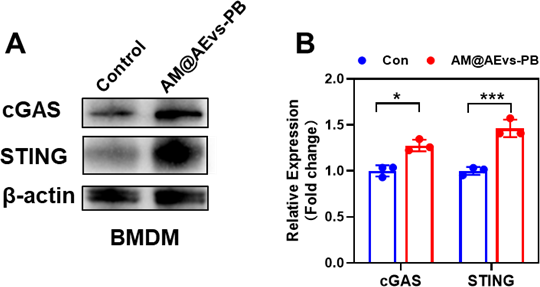


**Fig. S19.** Western blot analysis of the expression levels of STING pathway-related proteins in BMDM treated with AM@AEvs-PB for 24 h. **B)** Corresponding statistical analysis histograms of cGAS and STING (n = 3). Differences with *P* < 0.05 (*), *P* < 0.01 (**) or *P*

< 0.001 (***) were considered statistically significant.

# Fig. S20.


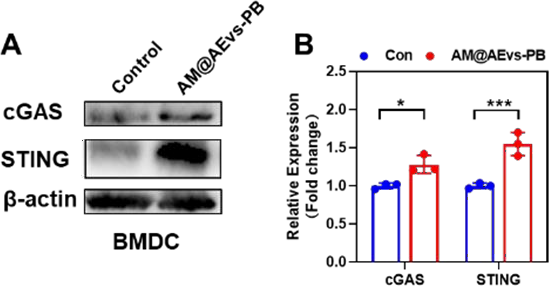


**Fig. S20.** Western blot analysis of the expression levels of STING pathway-related proteins in BMDC treated with AM@AEvs-PB for 24 h. **B)** Corresponding statistical analysis histograms of cGAS and STING (n = 3). Differences with *P* < 0.05 (*), *P* < 0.01 (**) or *P*

< 0.001 (***) were considered statistically significant.

# Fig. S21.


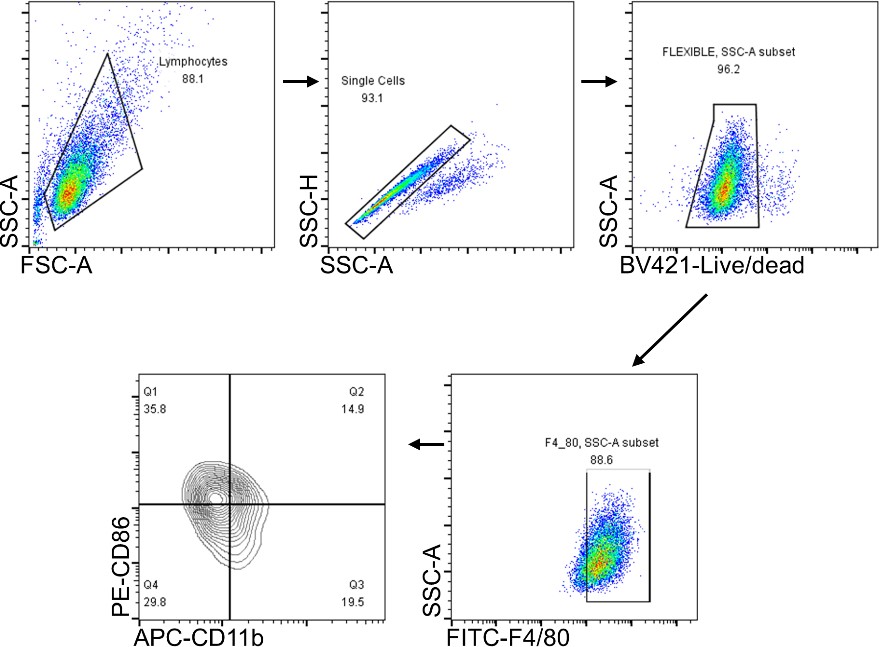


**Fig. S21.** Gating strategy to determine the percentages of BMDMs (F4/80^+^ CD11b^+^ CD86^+^).

# Fig. S22.


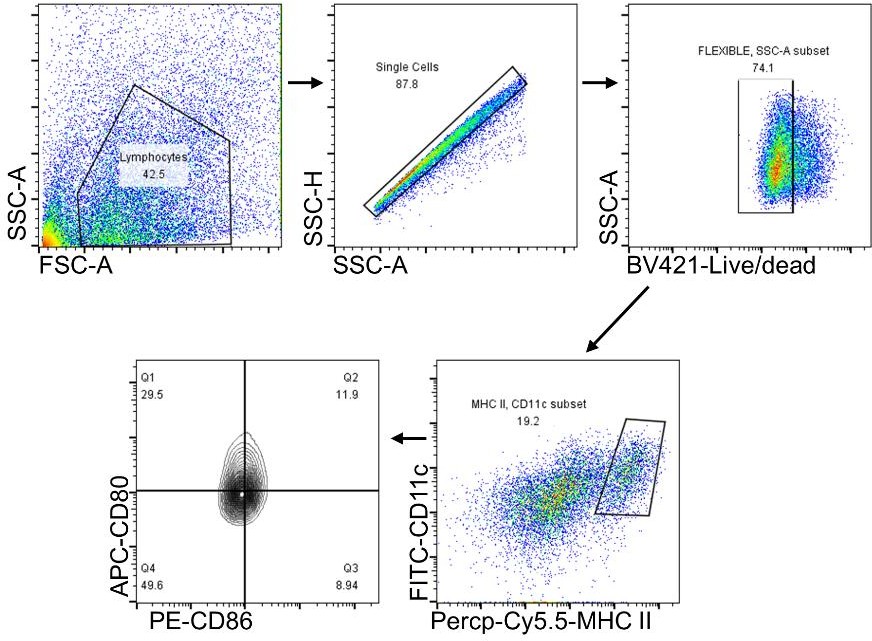


**Fig. S22.** Gating strategy to determine the percentages of matured BMDCs (MHC II^+^ CD11c^+^ CD80^+^ CD86^+^).

# Fig. S23.


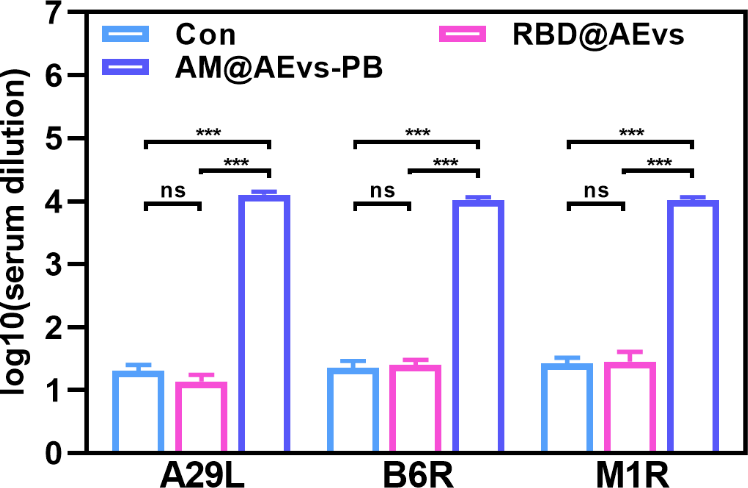


**Fig. S23.** MPV-related specific titers of different kind of mice (Balb/c and C57) after immunizing with AM@AEvs-PB. (n=3; ns: no significant; ***: *P<0.001*).

#
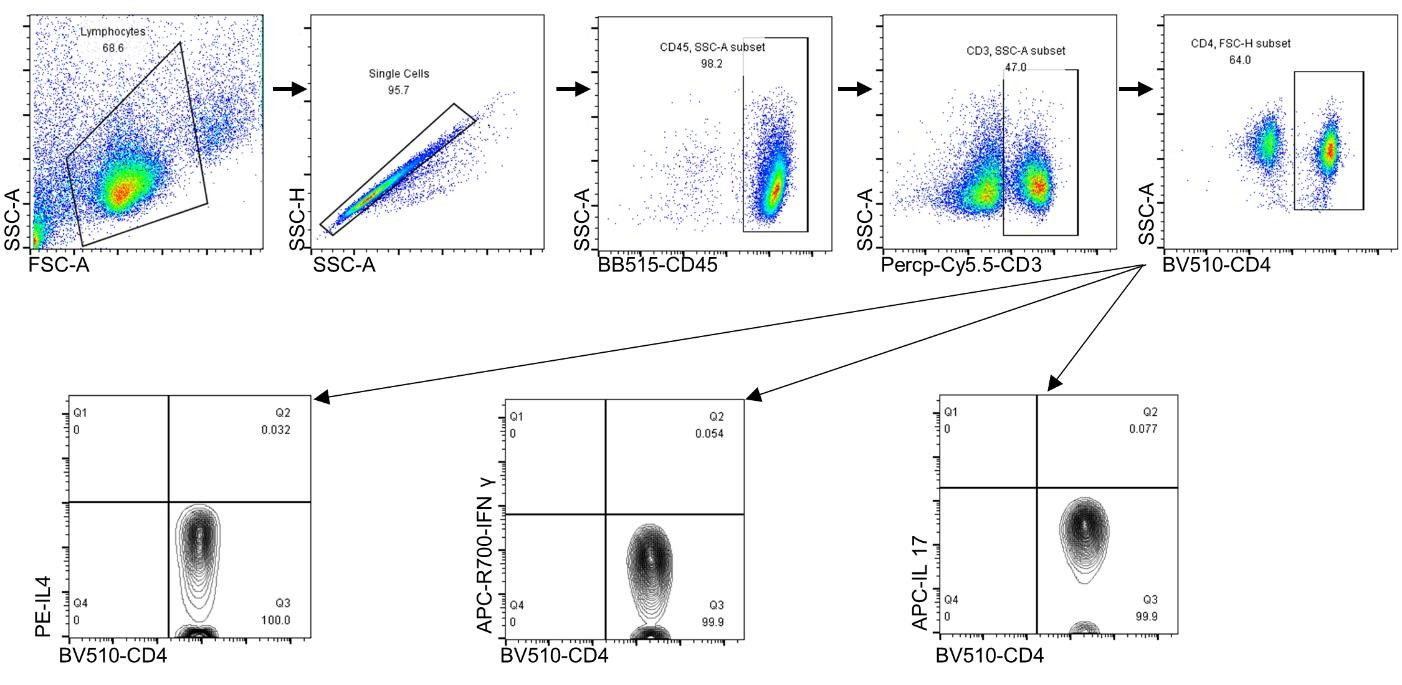
Fig. S24.

**Fig. S24.** Gating strategy to determine the percentages of T cells (CD45^+^ CD3^+^ CD4^+^).

#
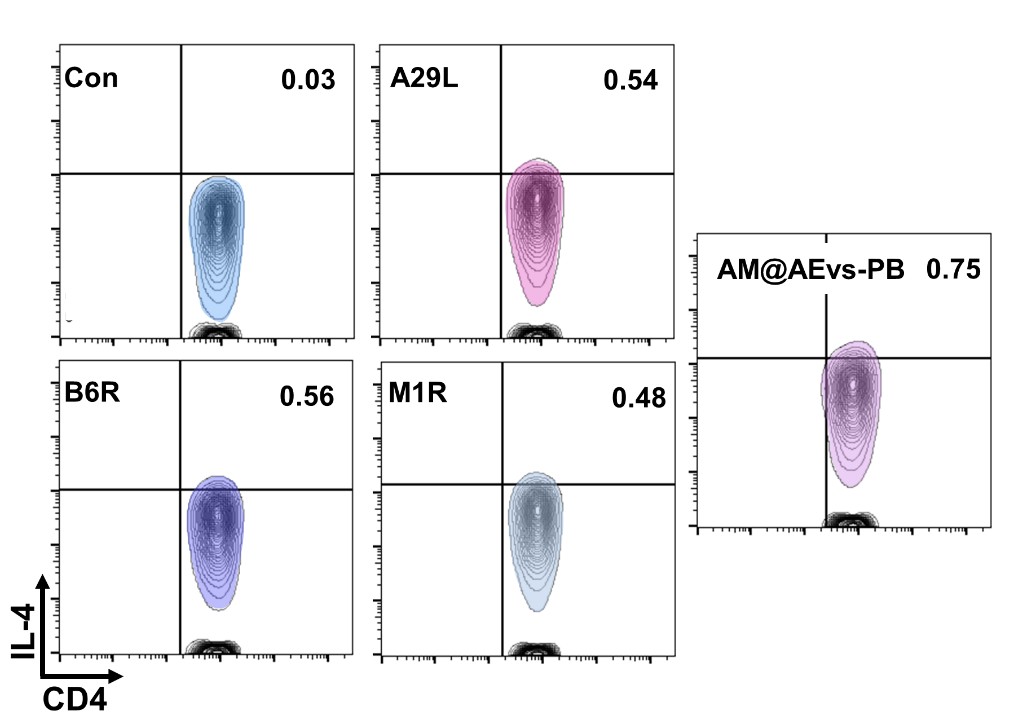
Fig. S25.

**Fig. S25.** The subpopulation of antigen specific splenic IL-4^+^ CD4^+^ T cells of mice vaccinating with A29L, B6R, M1R and AM@AEvs-PB for three times.

#
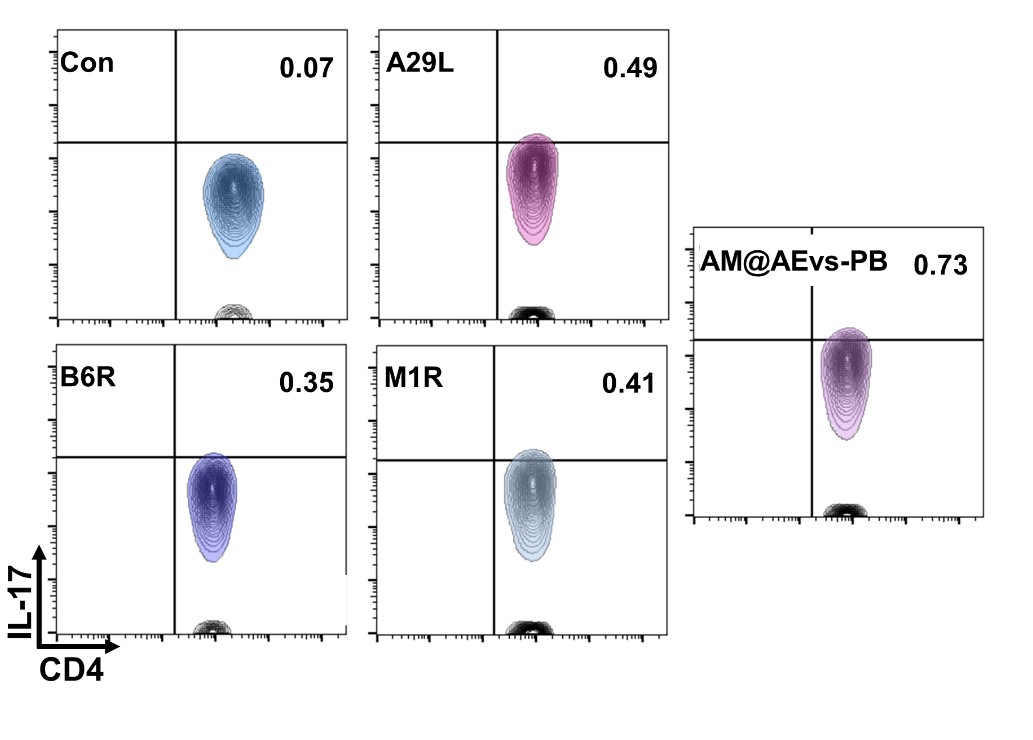
Fig. S26.

**Fig. S26.** The subpopulation of antigen specific splenic IL-17^+^ CD4^+^ T cells of mice vaccinating with A29L, B6R, M1R and AM@AEvs-PB for three times.

#
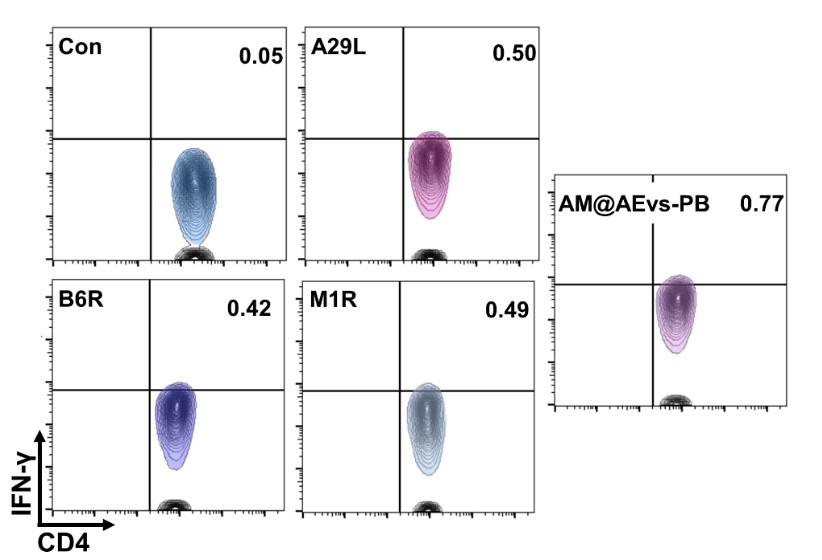
Fig. S27.

**Fig. S27.** The subpopulation of antigen specific splenic IFN-γ^+^ CD4^+^ T cells of mice vaccinating with A29L, B6R, M1R and AM@AEvs-PB for three times.

#
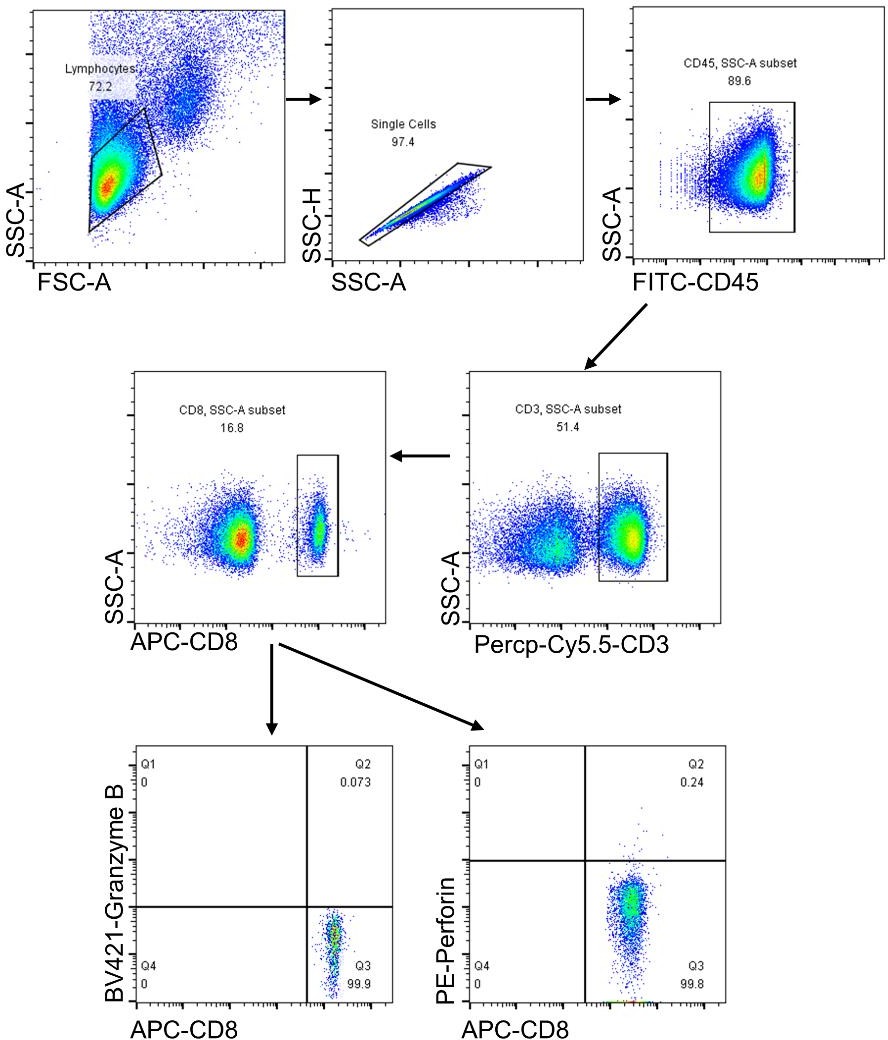
Fig. S28.

**Fig. S28.** Gating strategy to determine the percentages of T cells (CD45^+^ CD3^+^ CD8^+^).

# Fig. S29.


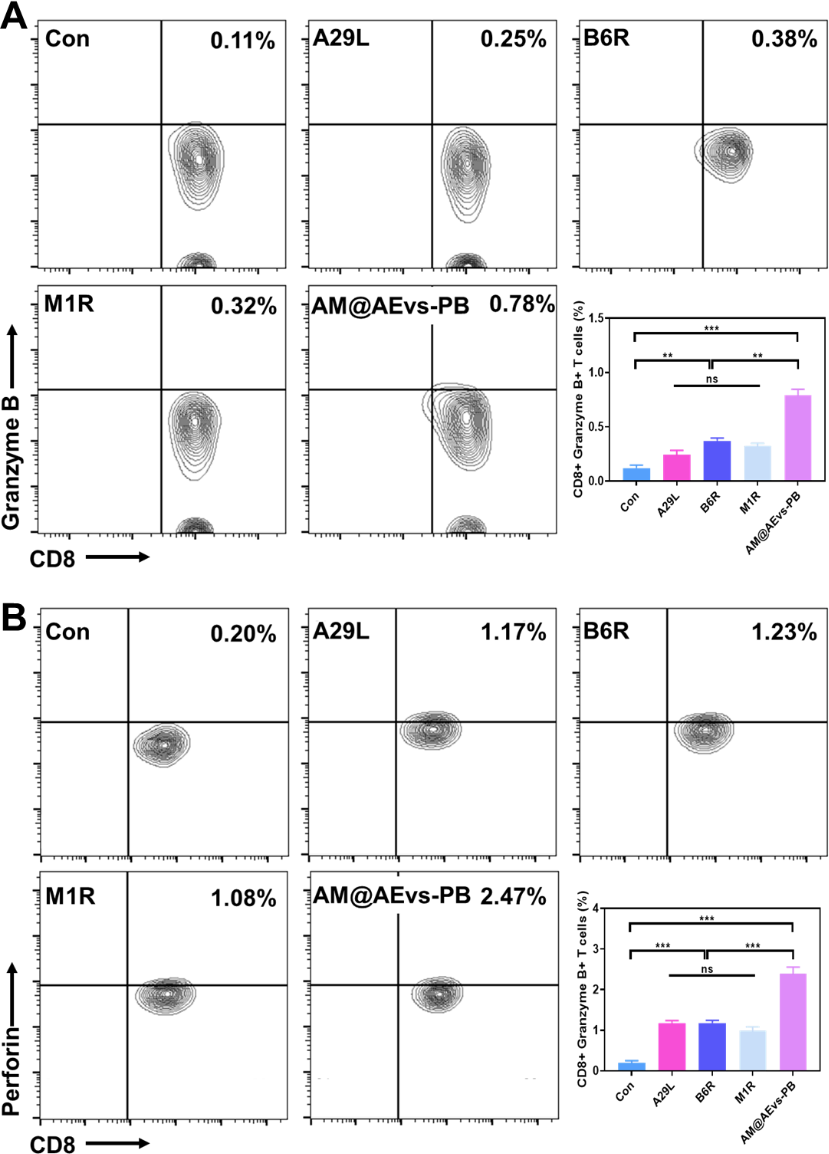


**Fig. S29.** Changes in the proportion of antigen specific splenic **A)** Granzyme B and **B)** perforin-secreting CD8^+^ T cells of mice vaccinating with A29L, B6R, M1R and AM@AEvs-PB for three times. (n=3; ns: no significance; **: *P<*0.01; **: *P<*0.001)

=

# Fig. S30.


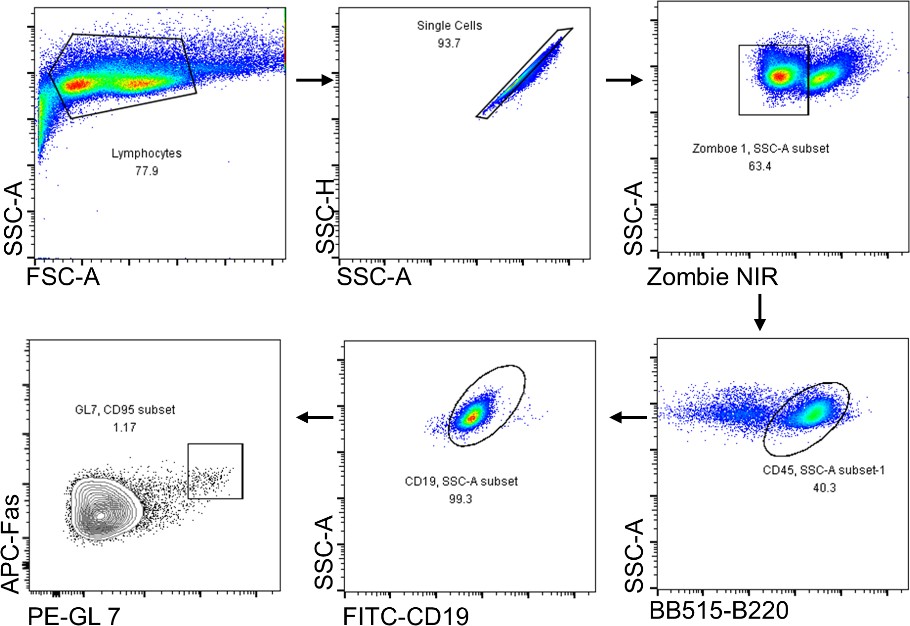


**Fig. S30.** Gating strategy to determine the percentages of activated GC B cells (B220^+^ CD19^+^ GL7^+^ Fas^+^).

#
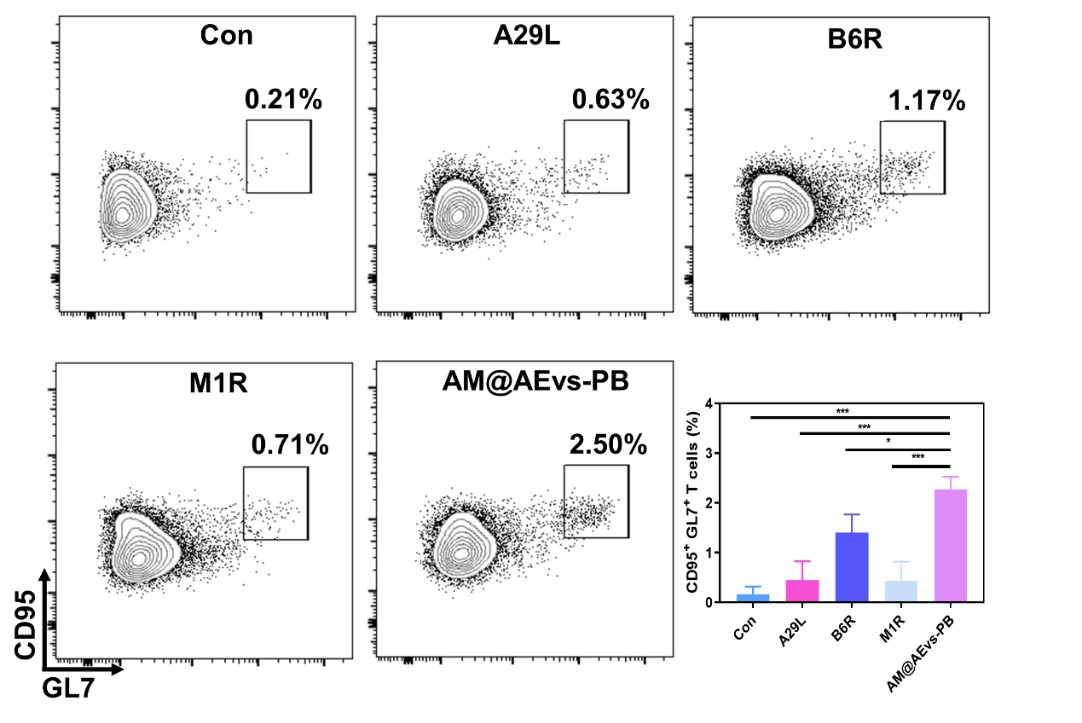
Fig. S31.

**Fig. S31.** The subpopulation of CD95^+^ GL7^+^ B cells (from CD19^+^ B cells) in lymph nodes of mice vaccinating with A29L, B6R, M1R and AM@AEvs-PB for three times. (n=5)

# Fig. S32.


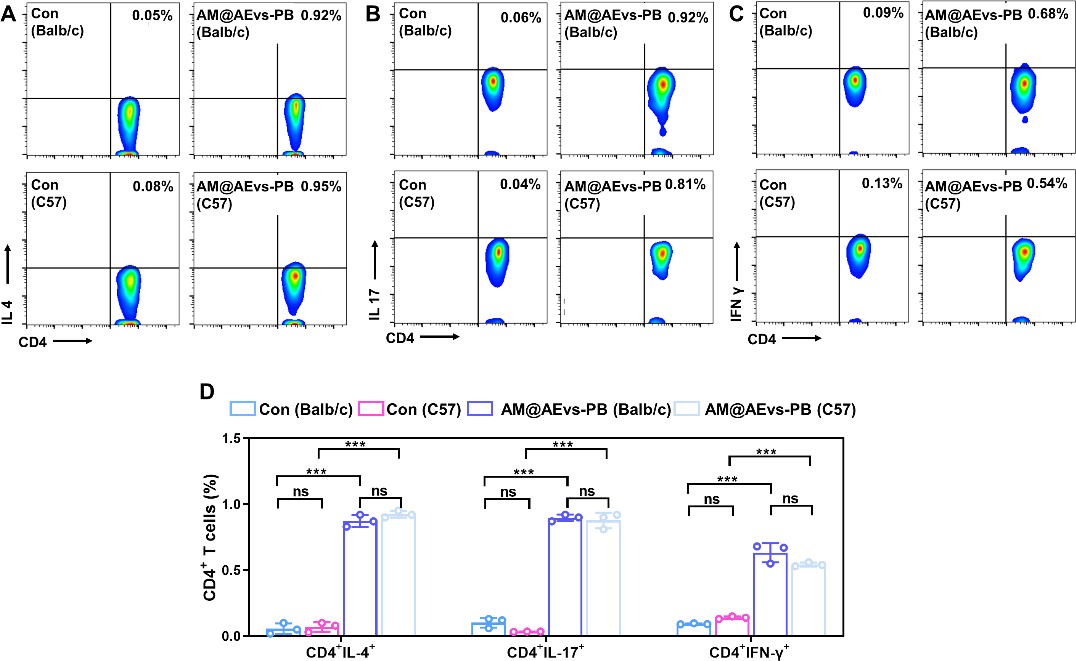


**Fig. S32.** The subpopulation of antigen specific splenic **A)** IL4^+^CD4^+^, **B)** IL17^+^ CD4^+^ and

**C)** IFNγ^+^CD4^+^T cells of Balb/c mice and C57 mice vaccinating with PBS and AM@AEvs- PB for three times. (n=3; ns: no significant; ***: *P<0.001*).

# Fig. S33.


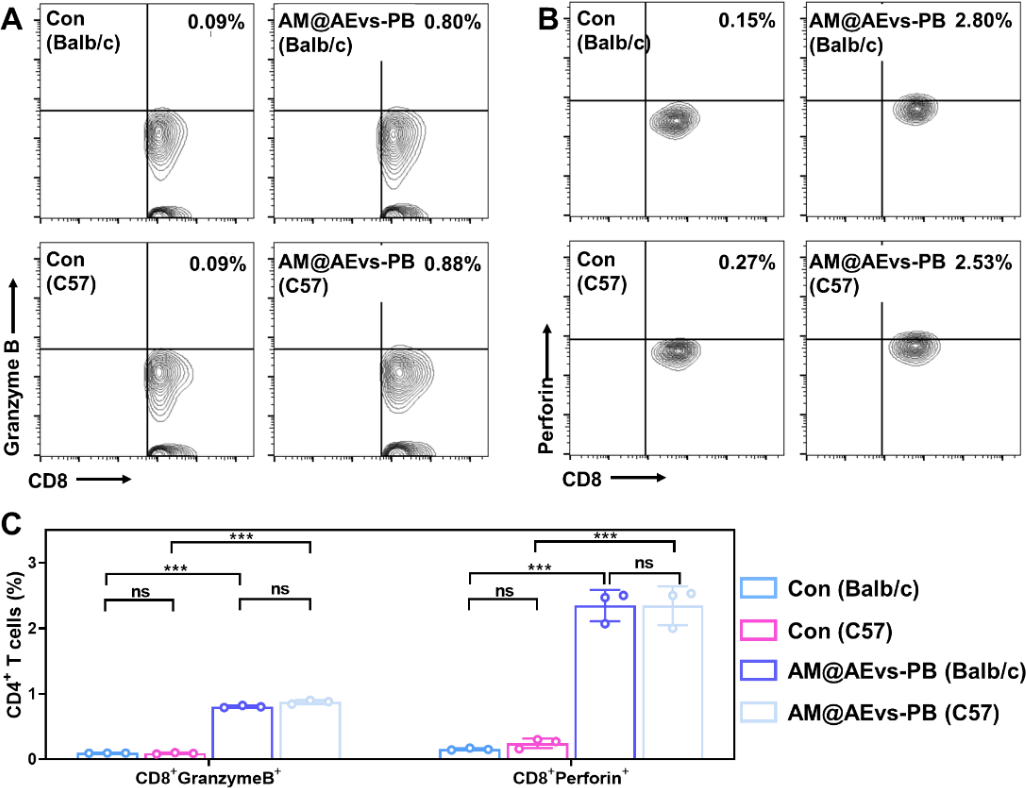


**Fig. S33.** The subpopulation of antigen specific splenic **A)** Granzyme B^+^CD8^+^ and **B)** Perforin^+^ CD8^+^ T cells of Balb/c mice and C57 mice vaccinating with PBS and AM@AEvs-PB for three times. (n=3; ns: no significant; ***: *P<0.001*).

# Fig. S34.


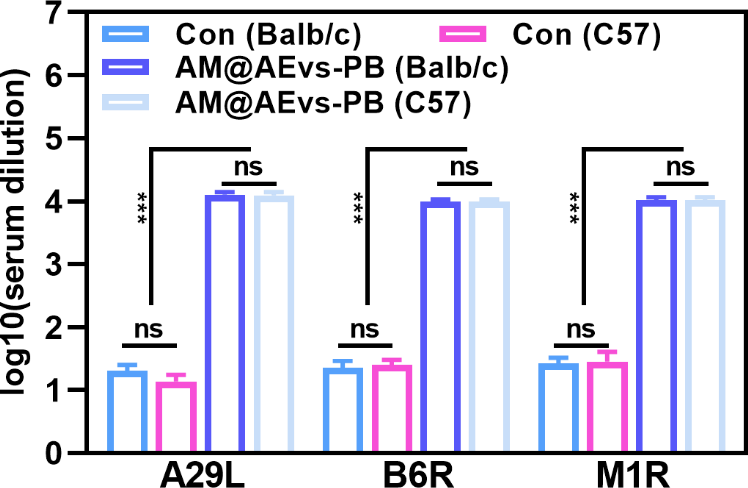


**Fig. 34.** MPV-related specific titers of different kind of mice (Balb/c and C57) after immunizing with AM@AEvs-PB. (n=3; ns: no significant; ***: *P<0.001*).

# Fig. S35.


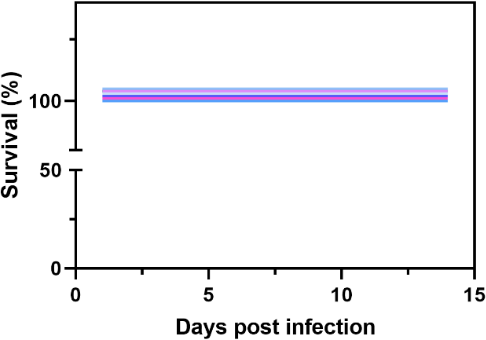


**Fig. S35.** The survial rate of mice immnunimized with PBS, Evs, A29L, B6R, M1R, AMB and AM@AEvs-PB after challenaged with VACA. (n=8)

# Fig. S36.


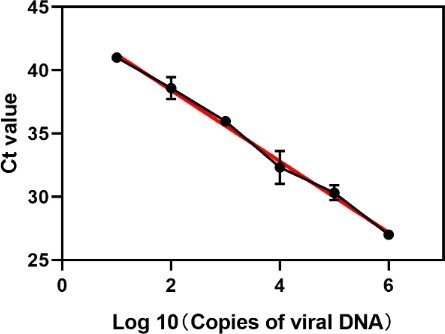


**Fig. S36.** The standard curve of viral mRNA ct value via qPCR.

#
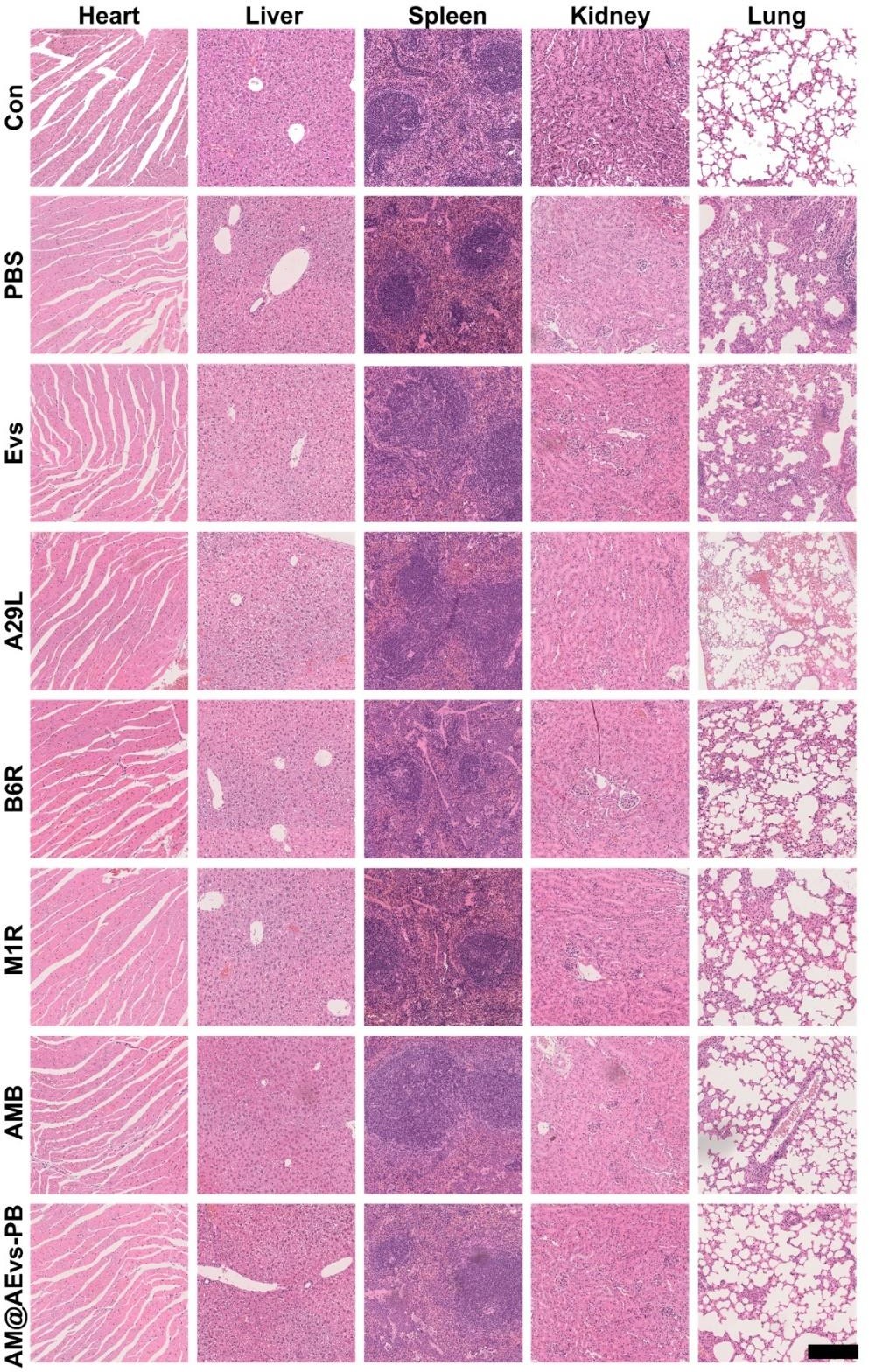
Fig. S37.

**Fig. S37.** The H&E staining of major organs of mice vaccinated with PBS, Evs, A29L, B6R, M1R, AMB and AM@AEvs-PB after challenged with VACA for 3 days (Scale bar: 200 μm).

#
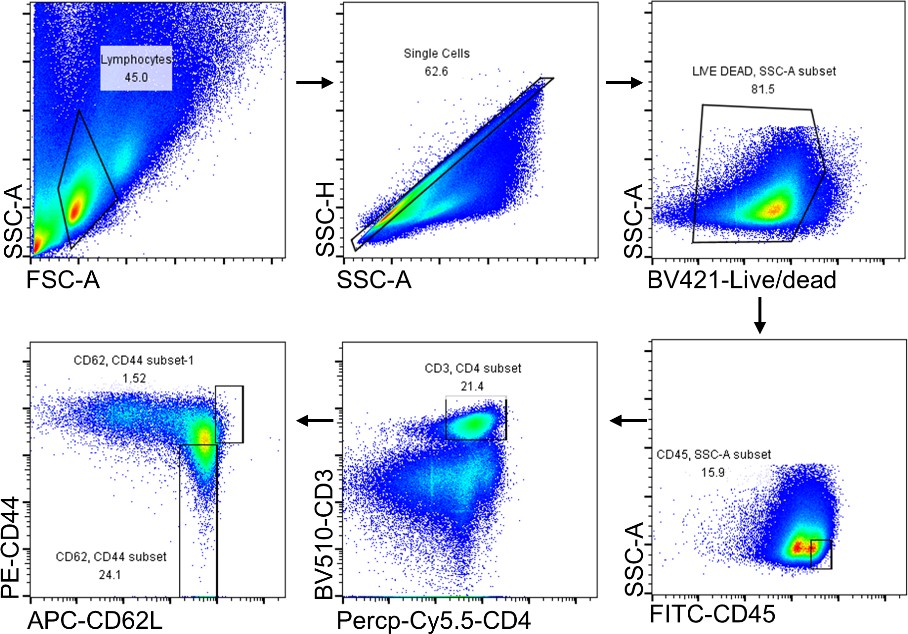
Fig. S38.

**Fig. S38.** Gating strategy to determine the percentages of T memory cells (CD45^+^ CD3^+^ CD4^+^ CD62L^+^ CD44^+^).

#
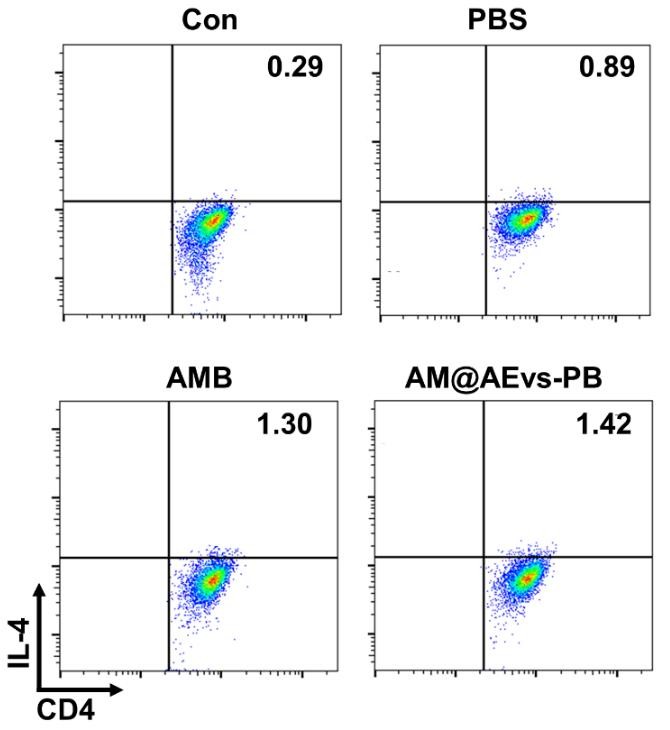
Fig. S39.

**Fig. S39.** The subpopulation of antigen specific splenic IL-4^+^ CD4^+^ T cells of vaccinated mice with PBS, AMB and AM@AEvs-PB 3 days after challenge.

#
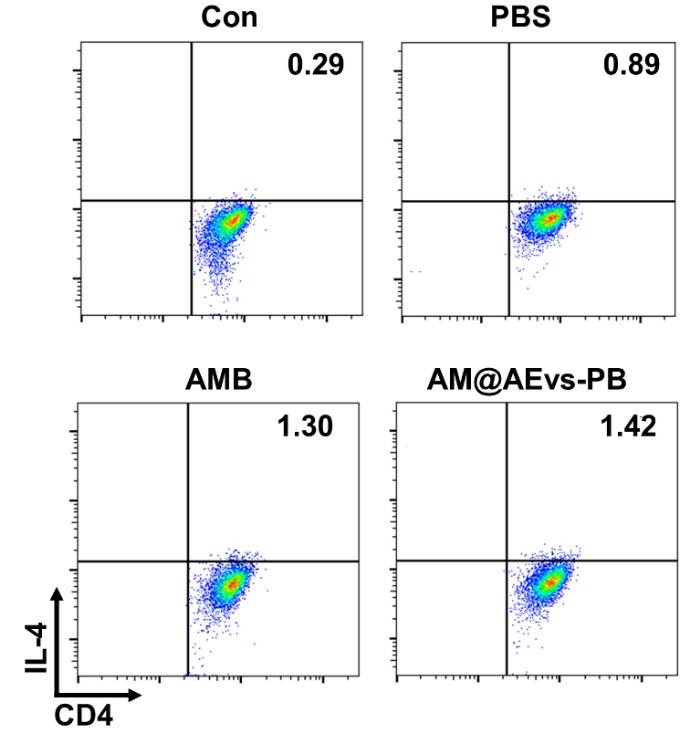
Fig. S40.

**Fig. S40.** The subpopulation of antigen specific splenic IL-17^+^ CD4^+^ T cells of vaccinated mice with PBS, AMB and AM@AEvs-PB 3 days after challenge.

#
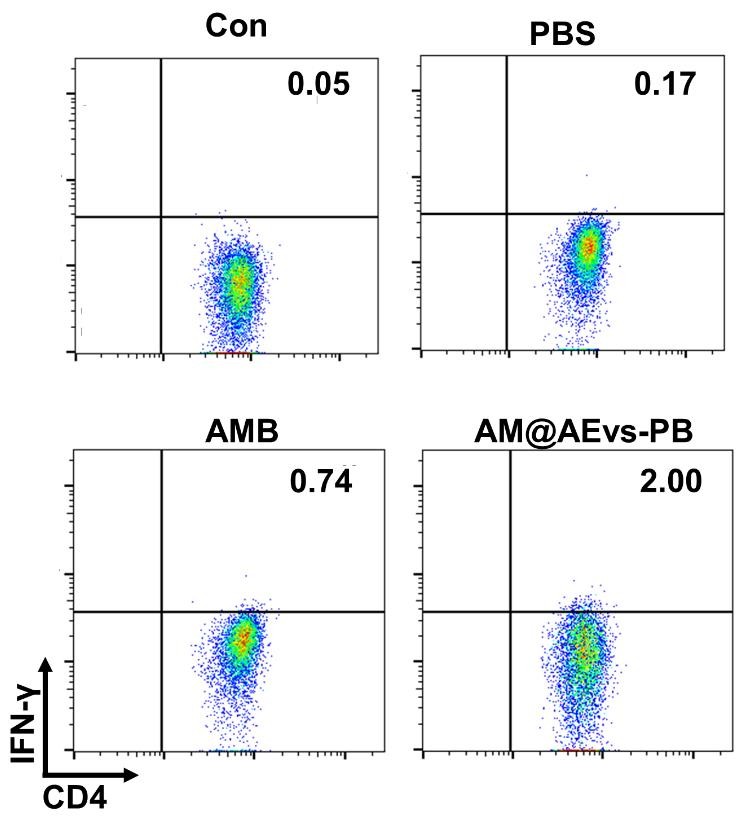
Fig. S41.

**Fig. S41.** The subpopulation of antigen specific splenic IFN-γ^+^ CD4^+^ T cells of vaccinated mice with PBS, AMB and AM@AEvs-PB 3 days after challenge.

# Fig. S42.


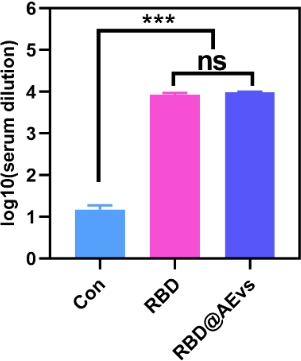


**Fig. S42.** RBD-specific titer after trice immunized with RBD and RBD@AEvs. (n=5; ns: no significant; ***: *P<0.001*)

# Fig. S43.


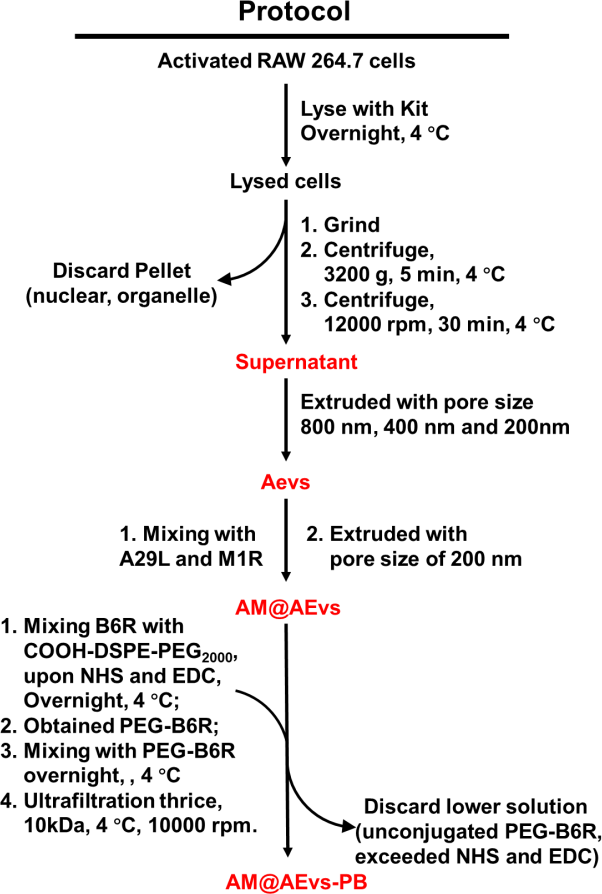


**Fig. S43.** Schematic illustration of the generation of AM@AEvs-PB.

# Table S1.

**Table S1. Up-regulated mRNA genes in Vax-treated RAW 264.7 cells detected by RNA-seq.**

| Gene (functional category) | Symbol | Fold | P value | P adj |
| --- | --- | --- | --- | --- |
| ***Viral protein interaction with cytokine and cytokine receptor*** | | | | |
| chemokine (C-C motif)  ligand 5 | Ccl5 | 3377.5 | pval | FDR |
| interleukin 6 | IL6 | 3633.6 | 5.1E-29 | 4.2E-25 |
| chemokine (C-X-C motif)  ligand 2 | Cxcl2 | 49.6 | 8.7E-24 | 3.3E-20 |
| chemokine (C-X-C motif)  ligand 3 | Cxcl3 | 170.5 | 1.7E-16 | 1.2E-13 |
| chemokine (C-X3-C motif)  ligand 1 | Cx3cl1 | 69.2 | 1.5E-13 | 5.5E-11 |
| tumor necrosis factor receptor superfamily member  1b | Tnfrsf1b | 12.7 | 6.4E-12 | 1.6E-09 |
| chemokine (C-C motif)  receptor 1 | Ccr1 | 12.1 | 2.2E-08 | 2.2E-06 |
| interleukin 2 receptor beta  chain | Il2rb | 315.8 | 6.0E-08 | 5.4E-06 |
| chemokine (C-C motif)  ligand 3 | Ccl3 | 9.1 | 3.2E-07 | 2.3E-05 |
| chemokine (C-C motif)  receptor 7 | Ccr7 | 16.5 | 8.5E-07 | 5.3E-05 |
| chemokine (C-X-C motif)  ligand 11 | Cxcl11 | 22.5 | 1.2E-06 | 6.9E-05 |
| chemokine (C-X-C motif)  receptor 5 | Cxcr5 | 8.5 | 2.3E-06 | 1.2E-04 |
| interleukin 2 receptor alpha  chain | Il2ra | 177.3 | 9.8E-06 | 4.4E-04 |
| chemokine (C-C motif)  ligand 2 | Ccl2 | 161.0 | 1.4E-05 | 5.9E-04 |
| chemokine (C-X-C motif)  ligand 10 | Cxcl10 | 6.5 | 5.3E-05 | 1.8E-03 |
| colony stimulating factor 1  (macrophage) | Csf1 | 4.9 | 9.3E-05 | 2.9E-03 |
| chemokine (C-C motif)  ligand 25 | Ccl25 | 4.1 | 1.5E-04 | 4.4E-03 |
| ***Toll-like receptor signaling pathway*** | | | | |
| interferon regulatory factor 7 | Irf7 | 172.4 | 1.7E-18 | 2.0E-15 |
| interleukin 1 beta | Il1b | 209.5 | 8.7E-13 | 2.5E-10 |
| FBJ osteosarcoma oncogene | Fos | 12.2 | 1.1E-07 | 9.1E-06 |
| toll-like receptor 9 | Tlr9 | 11.6 | 4.0E-07 | 2.7E-05 |
| secreted phosphoprotein 1 | Spp1 | 7.3 | 7.9E-06 | 0.0004 |
| signal transducer and  activator of transcription 1 | Stat1 | 4.6 | 3.7E-04 | 9.4E-03 |
| lymphocyte antigen 96 | Ly96 | 3.9 | 2.1E-03 | 3.9E-02 |
| ***TNF signaling pathway*** |  |  |  |  |

| prostaglandin-endoperoxide  synthase 2 | Ptgs2 | 7.8 | 6.3E-20 | 1.2E-16 |
| --- | --- | --- | --- | --- |
| leukemia inhibitory factor | Lif | 7.8 | 5.5E-19 | 7.2E-16 |
| tumor necrosis factor alpha- | Tnfaip3 | 7.9 | 1.4E-08 | 1.5E-06 |
| induced protein 3 |  |  |  |  |
| interleukin 15 | Il15 | 3.9 | 2.5E-05 | 1.0E-03 |
| endothelin 1 | Edn1 | 7.1 | 2.4E-04 | 6.5E-03 |
| jagged 1 | Jag1 | 19.7 | 5.7E-04 | 1.4E-02 |
| Fas (TNF receptor | Fas | 4.3 |  |  |
| superfamily member 6) |  |  | 1.2E-03 | 2.5E-02 |
| matrix metallopeptidase 9 | Mmp9 | 4.8 | 2.3E-03 | 4.1E-02 |
| suppressor of cytokine | Socs3 | 3.7 | 1.4E-07 | 1.1E-05 |
| signaling 3 |  |  |  |  |
| ***Cytokine-cytokine receptor interaction*** | | | | |
| colony stimulating factor 3 | Csf3 | 2125.0 | 1.3E-22 | 3.7E-19 |
| (granulocyte) |  |  |  |  |
| interleukin 33 | Il33 | 5550.8 | 2.7E-18 | 3.0E-15 |
| interleukin 11 | Il11 | 537.6 | 1.0E-16 | 8.0E-14 |
| interleukin 1 alpha | Il1a | 76.4 | 1.4E-12 | 4.1E-10 |
| interleukin 1 receptor |  | 48.3 | 2.3E-11 | 4.9E-09 |
| antagonist | Il1rn |  |  |  |
| tumor necrosis factor |  | 83.0 | 2.7E-11 | 5.7E-09 |
| receptor superfamily member |  |  |  |  |
| 13b | Tnfrsf13b |  |  |  |
| growth differentiation factor |  | 22.3 | 1.2E-09 | 1.7E-07 |
| 15 | Gdf15 |  |  |  |
| oncostatin M | Osm | 20.0 | 1.2E-09 | 1.7E-07 |
| tumor necrosis factor |  | 17.2 | 5.7E-09 | 6.8E-07 |
| receptor superfamilymember |  |  |  |  |
| 9 | Tnfrsf9 |  |  |  |
| transforming growth |  | 450.4 | 4.4E-08 | 3.7E-19 |
| factorbeta 3 | Tgfb3 |  |  |  |
| inhibin beta-A | Inhba | 277.4 | 2.4E-06 | 1.3E-04 |
| cardiotrophin-like cytokine |  | 29.4 | 1.7E-05 | 7.1E-04 |
| factor 1 | Clcf1 |  |  |  |
| interleukin 36G | Il36g | 141.2 | 2.0E-04 | 5.6E-03 |
| tumor necrosis factor | Tnfrsf12a | 4.3 | 6.6E-04 | 1.5E-02 |
| receptor superfamily member |  |  |  |  |
| 12a |  |  |  |  |
| colony stimulating factor 2 | Csf2rb2 | 4.0 | 1.5E-03 | 2.9E-02 |
| receptor beta 2 low-affinity |  |  |  |  |
| (granulocyte-macrophage) |  |  |  |  |
| ***Chemokine signaling pathway*** |  |  |  |  |
| chemokine (C-C motif) ligand | Ccl5 | 3377.5 | 5.1E-29 | 4.2E-25 |
| 5 |  |  |  |  |
| chemokine (C-C motif) ligand | Ccl22 | 19851.7 | 8.7E-24 | 3.3E-20 |
| 22 |  |  |  |  |
| chemokine (C-X-C motif) | Cxcl2 | 49.6 | 1.5E-13 | 5.5E-11 |
| ligand 2 |  |  |  |  |

| chemokine (C-X-C motif)  ligand 3 | Cxcl3 | 170.5 | 6.4E-12 | 1.6E-09 |
| --- | --- | --- | --- | --- |
| Rous sarcoma oncogene | Src | 24.2 | 1.4E-09 | 2.0E-07 |
| chemokine (C-X3-C motif)  ligand 1 | Cx3cl1 | 69.2 | 2.2E-08 | 2.2E-06 |
| chemokine (C-C motif)  receptor 1 | Ccr1 | 12.1 | 3.2E-07 | 2.3E-05 |
| guanine nucleotide binding  protein (G protein) gamma 8 | Gng8 | 11.5 | 9.1E-07 | 5.6E-05 |
| chemokine (C-C motif) ligand  3 | Ccl3 | 9.1 | 1.2E-06 | 6.9E-05 |
| chemokine (C-C motif)  receptor 7 | Ccr7 | 16.5 | 2.3E-06 | 1.2E-04 |
| chemokine (C-X-C motif)  ligand 11 | Cxcl11 | 22.5 | 9.8E-06 | 4.4E-04 |
| chemokine (C-X-C motif)  receptor 5 | Cxcr5 | 8.5 | 1.4E-05 | 5.9E-04 |
| predicted gene 4356 | Gm4356 | 26.8 | 2.9E-05 | 1.1E-03 |
| chemokine (C-C motif) ligand  2 | Ccl2 | 161.0 | 9.3E-05 | 2.9E-03 |
| chemokine (C-X-C motif)  ligand 10 | Cxcl10 | 6.5 | 1.5E-04 | 4.4E-03 |
| breast cancer anti-estrogen  resistance 1 | Bcar1 | 6.9 | 3.3E-04 | 8.6E-03 |
